# Monomeric ZRANB3 drives replication fork reversal

**DOI:** 10.1093/nar/gkag1034

**Published:** 2026-11-03

**Authors:** Yen-Ju Chen, Yu-Hui Chiu, Chieh-Kai Liu, Hsuan Hsu, Kuan-Chi Wang, Guan-Ting Lian, Yi-An Chen, Sheng-Wei Lin, Hsin-Yung Yen, Hung-Wen Li, Peter Chi

**Affiliations:** Institute of Biochemical Sciences, National Taiwan University, Taipei 106, Taiwan; Institute of Biochemical Sciences, National Taiwan University, Taipei 106, Taiwan; Department of Chemistry, National Taiwan University, Taipei 106, Taiwan; Department of Chemistry, National Taiwan University, Taipei 106, Taiwan; Department of Chemistry, National Taiwan University, Taipei 106, Taiwan; Institute of Biochemical Sciences, National Taiwan University, Taipei 106, Taiwan; Institute of Biological Chemistry, Academia Sinica, Taipei 106, Taiwan; Chemical Biology and Molecular Biophysics Program, Taiwan International Graduate Program, Academia Sinica, Taipei 106, Taiwan; Institute of Biological Chemistry, Academia Sinica, Taipei 106, Taiwan; Institute of Biological Chemistry, Academia Sinica, Taipei 106, Taiwan; Institute of Biological Chemistry, Academia Sinica, Taipei 106, Taiwan; Department of Chemistry, National Taiwan University, Taipei 106, Taiwan; Institute of Biochemical Sciences, National Taiwan University, Taipei 106, Taiwan; Institute of Biological Chemistry, Academia Sinica, Taipei 106, Taiwan

## Abstract

Replication fork reversal is a central response to replication stress, yet how a SNF2 translocase coordinates the distinct mechanical steps required for this reaction remains elusive. Here, we define the mechanism by which ZRANB3 catalyzes fork reversal using biochemical, biophysical, and single-molecule approaches. ZRANB3 binds fork DNA as a monomer at the junction, establishing the minimal functional unit required for activity. Mechanistically, ZRANB3 combines ATP-dependent localized duplex opening with ATP-independent strand annealing to facilitate four-way junction formation, followed by directional branch migration that supports long-range fork reversal. Single-molecule fluorescence analyses further demonstrate that monomeric ZRANB3 is sufficient to drive fork reversal. Together, these findings define how ZRANB3 integrates distinct DNA remodeling activities within a single enzyme to catalyze fork reversal.

## Graphical Abstract

**Graphical Abstract:**
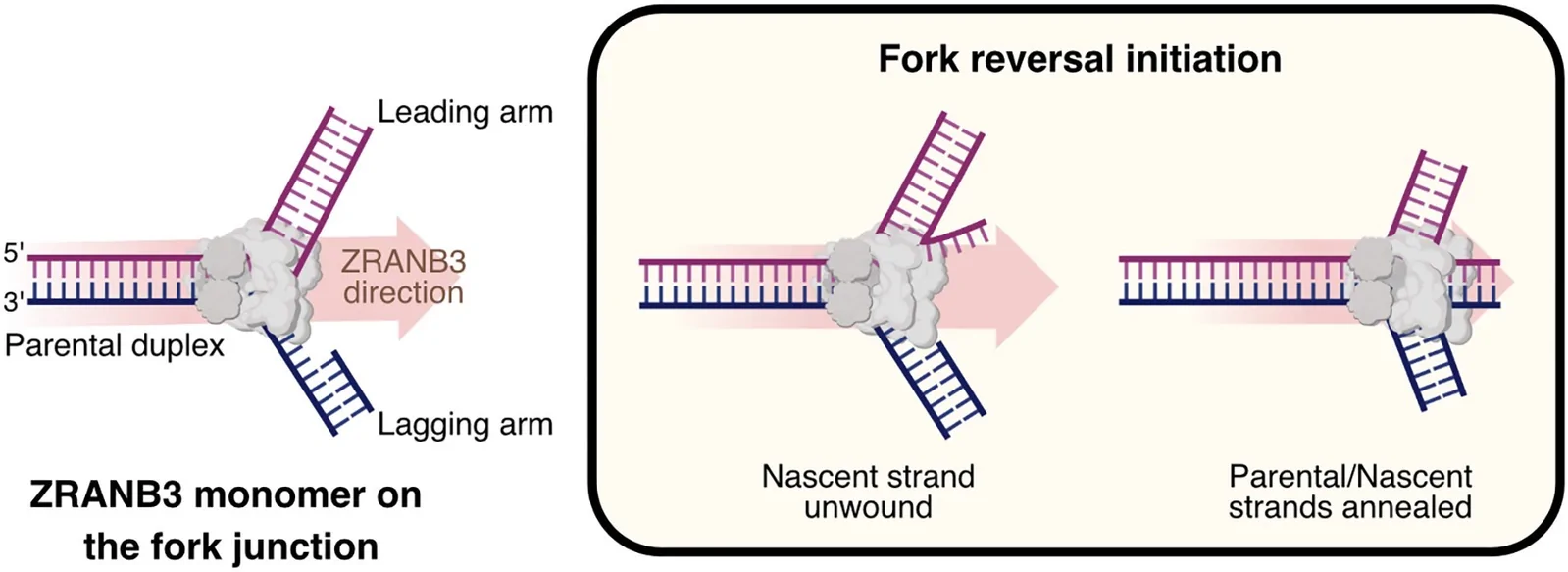

## Introduction

Replication stress causes replication forks to slow or stall, posing a significant threat to genome stability [1, 2]. Stalled replication forks frequently arise in response to endogenous and exogenous DNA damage, including through interstrand crosslinks, DNA–protein complexes, repetitive DNA sequences forming secondary structures, and collisions with the transcription machinery. These impediments not only jeopardize cell viability, but they also contribute to the development of various pathological conditions, such as cancer, by compromising the fidelity of DNA replication [2, 3]. To mitigate these challenges, eukaryotic cells have evolved multiple pathways to overcome replication stress, including PrimPol-mediated repriming, translesion DNA synthesis, template switching, and fork reversal, each with distinct trade-offs in maintaining replication fidelity and genomic integrity [3, 4]. Fork reversal converts a replication fork into a four-way “chicken foot” structure, which has emerged as a critical protective mechanism that limits single-stranded DNA (ssDNA) accumulation and facilitates DNA lesion bypass and fork restart. This process is physiologically significant as it helps preserve genome integrity and prevents the accumulation of mutations that can lead to cellular dysfunction and diseases [4, 5].

In higher eukaryotes, fork reversal is orchestrated by the specialized ATP-dependent SNF2-family DNA translocases SMARCAL1, ZRANB3, and HLTF, along with other factors including FBH1 and RAD51 [6–10]. iPOND (isolation of proteins on nascent DNA) [11, 12] and SIRF (*in situ* protein interaction with nascent DNA replication forks) [13–15] assays have provided compelling evidence for robust accumulation of SMARCAL1, ZRANB3, and HLTF at stalled replication forks, underscoring their pivotal role in responding to replication stress. Electron microscopy studies have shown that depletion of any of these DNA translocases markedly reduces the number of reversed forks, as evidenced by a significant reduction in “chicken foot” structures [9, 16–19]. These findings have not only established that SMARCAL1, ZRANB3, and HLTF catalyze fork reversal, but they also highlight the nonredundant functions of these proteins in maintaining replication fork stability under genotoxic stress [17, 19–21].

Biochemical analyses have revealed that the aforementioned SNF2-family translocases exhibit distinct fork remodeling activities [11, 22–26]. SMARCAL1, HLTF, and ZRANB3 all share a conserved two-lobed RecA-type ATPase domain that drives ATP hydrolysis and provides the motor function required for DNA remodeling [6]. However, their accessory domains confer distinct functions and modes of recruitment onto stalled replication forks. For instance, SMARCAL1 is recruited via an RPA2 interaction motif and harnesses its dual HARP domains to promote ATP-dependent fork reversal primarily through coordinated strand annealing and branch migration [22, 23, 27]. In contrast, HLTF features a HIRAN domain that recognizes 3′ ssDNA ends at stalled forks [11] and a RING domain that facilitates PCNA polyubiquitination [28, 29], thereby linking its remodeling activity to template switching events [30]. Though ZRANB3 shares significant core homology with SMARCAL1, it is uniquely recruited through PCNA interaction mediated by its PIP box, APIM motif, and NPL4 zinc finger [24, 31]. ZRANB3 also possesses a distinctive HNH nuclease domain that imparts structure-specific endonuclease activity, enabling it to process and remodel DNA lesions at stalled forks [32, 33]. Together, these studies have not only highlighted the nonredundant roles of these DNA translocases in fork reversal, but they have also provided critical insights into the distinct mechanisms that underpin the action of these enzymes in the replication stress response.

Mechanistically, fork reversal is thought to involve a highly coordinated reaction in which nascent strands are displaced from their parental templates, the parental strands reanneal, and the nascent strands subsequently reanneal to form a regressed arm [16]. The SNF2-family proteins SMARCAL1, ZRANB3, and HLTF have been documented in catalyzing this process and are generally described as double-stranded DNA (dsDNA) translocases, although their capacity to directly unwind duplex DNA remains unclear. Notably, unwinding has been observed in assays using fork substrates with homologous arms (Fig. 1A, top panel). In contrast, these enzymes fail to exhibit detectable helicase activity on partial duplex substrates or fork DNA containing heterologous arms (Fig. 1A, bottom panel) [25, 34, 35]. These observations have led to the suggestion that any strand-separating activity of these motors, if present, may be limited, transient, or tightly coupled to annealing [22, 25, 34–36].

Insights from bacterial and yeast homologs indicate that DNA unwinding is likely to be an integral step of the fork reversal process. For example, the *Escherichia coli* RecG helicase actively unwinds fork DNA strands with heterologous arm at replication fork junctions through its distinctive “wedge” domain [37], though the precise role of RecG *in vivo* remains a subject of debate [38]. Similarly, *Saccharomyces cerevisiae* Rad5, a homolog of human HLTF, has been shown to promote fork reversal via leading strand unwinding [39]. These observations prompted us to reexamine whether replication fork reversal by translocases entails a DNA strand separation activity. Moreover, structural analysis and biochemical studies collectively establish that RecG drives fork reversal as a functional monomeric motor [37, 40]. However, the functional oligomeric states of mammalian SNF2 translocases upon engaging replication fork DNA remain unclear. Here, we sought to address these fundamental mechanistic questions by defining how ZRANB3 engages and remodels replication forks during fork reversal.

## Materials and methods

### Cloning, expression, and purification of recombinant proteins

The translocases used in this study were cloned into pcDNA3.4 vector to generate an N-terminal (His)_6_-Flag fusion construct. For Alexa488 labeling, the SNAP tag was cloned between the (His)_6_-Flag and the ZRANB3 sequence. Mutant variants of ZRANB3 (K163D and H1021A) were generated via site-directed mutagenesis, and all expression plasmids were verified by sequencing.

Expi293F human cells were transfected with the plasmids according to the instruction manual from the ExpiFectamine 293 kit (Thermo Fisher). Cells were harvested by centrifugation 48 h after transfection.

Protein purification was performed at 4°C. Cell pellets were resuspended in Buffer A [25 mM Tris–HCl, pH 7.5, 10% glycerol, 500 mM KCl, 0.01% Igepal-CA-630, 0.5 mM ethylenediaminetetraacetic acid (EDTA), 10 mM ATP, and 10 mM MgCl₂] supplemented with 1 mM β-mercaptoethanol and protease inhibitors (2 mM phenylmethylsulfonyl fluoride (PMSF), 2 mM benzamidine, and 1 μg/ml each of aprotinin, chymostatin, leupeptin, and pepstatin A). Cells were lysed by sonication, and the lysate was clarified by centrifugation at 100,000 × *g* for 1 h. The supernatant containing the target protein was incubated with Ni²⁺-NTA agarose resin (QIAGEN) for 2 h. The resin was washed sequentially with Buffer A containing 10 mM imidazole, followed by Buffer B (25 mM Tris–HCl, pH 7.5, 10% glycerol, 300 mM KCl, 0.01% Igepal-CA-630, 0.5 mM EDTA, 10 mM ATP, and 10 mM MgCl₂) containing 20 mM imidazole. The translocase was then eluted with Buffer B supplemented with 200 mM imidazole. The elution was further incubated with anti-Flag M2 affinity agarose resin (Sigma Aldrich), washed with Buffer B, and eluted with Buffer B containing 100 μg/ml of 3 × Flag peptide. Finally, the eluted protein was concentrated and subjected to size-exclusion chromatography using a Superdex 200 Increase 10/300 GL column (GE Healthcare) equilibrated with Buffer C (25 mM Tris–HCl, pH 7.5, 10% glycerol, 0.01% Igepal-CA-630, 1 mM β-mercaptoethanol, and 0.5 mM EDTA) supplemented with 300 mM KCl. The fractions containing the purified protein were collected, concentrated, and stored at –80°C.

The same purification protocol was applied to both wild-type and mutant translocases.

### *In vitro* fluorescence labeling of SNAP-ZRANB3 protein

SNAP-ZRANB3 (5 μM) was incubated with 10 μM SNAP-surface Alexa Fluor 488 (New England Biolabs, S9129S) in 50 μl buffer C supplemented with 300 mM KCl and 1 mM dithiothreitol (DTT) for 16 h at 4 °C. The reaction mixture was then centrifuged at 6,000 × *g* at 4 °C using an Amicon Ultra-4 30K centrifugal tube (Millipore) to remove the excess fluorescent dye and concentrate the labeled ZRANB3. The labeled ZRANB3 was divided into small aliquots and stored at −80 °C.

### Labeling efficiency determination

The labeling efficiency of SNAP-tagged proteins was determined spectrophotometrically. A standard curve of SNAP–Alexa Fluor 488 (SNAP-AF488) was prepared in buffer C supplemented with 150 mM KCl by measuring absorbance at 494 nm over a range of known dye concentrations. The absorbance of labeled protein samples at 494 nm was then used to determine dye concentration from the standard curve. Protein concentrations were quantified by sodium dodecyl sulphate (SDS)–PAGE densitometry using bovine serum albumin (BSA) as a standard. The resulting dye-to-protein ratio indicated a labeling efficiency of ∼90%.

### DNA substrates

The DNA substrates utilized in this study are listed in Supplementary Table S1, and the oligonucleotides used for their composition are provided in Supplementary Table S2. Fluorescently-labeled DNA oligonucleotides were purchased from Genomics.

For the fork reversal assay, leading gap DNA substrates were prepared as follows. Oligos A and sB were annealed at a 1:1.2 molar ratio in annealing buffer (50 mM Tris–HCl, pH 7.5, 10 mM MgCl₂, 100 mM NaCl, and 1 mM DTT) to form the upper strand of the fork substrate. In parallel, oligos D and C were annealed to form the bottom strand. Each mixture was heated to 80°C for 3 min, then held at 65°C for 30 min, and subsequently cooled to room temperature. The upper and bottom strands were then mixed in a 1:1 molar ratio and incubated at 37°C for 30 min. The annealed products were separated and purified from a 6% Tris–borate–EDTA (TBE) PAGE gel via electro-elution, concentrated using an Amicon Ultra-4 concentrator (Millipore, NMWL 30 kDa) at 7,000 × *g* at 4°C, and finally stored in Tris-EDTA (TE) buffer (10 mM Tris–HCl, pH 8.0, 0.5 mM EDTA). Lagging gap fork substrates, composed of oligos A, B, sC, and D, were prepared using the same procedure as for the leading gap substrate.

To prepare fork substrates for unwinding, footprinting, mass photometry, and native mass spectrometry (MS), the corresponding oligonucleotides were mixed at a 1:1 molar ratio in annealing buffer. The mixtures were heated to 80°C for 3 min, then incubated at 37°C for 30 min, and finally cooled to room temperature overnight. The products were separated and purified from a 10% TBE–PAGE gel via electro-elution, with subsequent processing identical to that described above for the fork reversal substrates.

### Fork reversal assay

In the fork reversal assay, 16 nM DNA substrate was incubated with the specified amounts of translocases in 10 μl of Buffer D (35 mM Tris–HCl, pH 7.5, 1 mM DTT, 0.1 μg/μl BSA, 50 mM KCl, 2 mM ATP, and 2 mM MgCl₂) at 37°C for 20 min. The reaction was terminated by adding 2.5 μl of termination buffer (with final 48 mM EDTA, 0.02% SDS, and 0.64 mg/ml proteinase K) and incubating at 37°C for 15 min. Next, 3 μl of loading buffer (20 mM Tris–HCl, pH 7.5, 50% glycerol) was added. Samples were then analyzed via 6% TBE–PAGE. The resulting gels were imaged using an Amersham™ Typhoon™ Biomolecular Imager (GE Healthcare) equipped with a Cy3 570BP20 560–580 nm filter, and the signal intensities of DNA species were quantified using Amersham™ ImageQuant™ software.

### Unwinding assay

The designated fork DNA (10 nM) was incubated with the specified amounts of translocases in 10 μl of Buffer D supplemented with 100 nM trap DNA at 37°C for 20 min. The reaction was stopped by adding 2.5 μl of termination buffer and incubating at 37°C for 15 min. Next, 3 μl of loading buffer was added, and the samples were analyzed by 10% TBE–PAGE. The resulting gels were imaged using an Amersham™ Typhoon™ Biomolecular Imager (GE Healthcare) equipped with Cy3 (570BP20; 560–580 nm) and Cy5 (670BP30; 655–685 nm) filters, and the signal intensities of DNA species were quantified using Amersham™ ImageQuant™ software. Unwinding activity was quantified by subtracting the signal of the no-protein control from that of the experimental samples.

### Single-stranded DNA annealing assay

In this assay, 3 nM of Cy3-labeled Oligo 8 was initially incubated with the indicated amount of ZRANB3 in Buffer D on ice. Subsequently, 3 nM of the complementary strand, Oligo 9, was added, and the mixture was incubated at 37°C for 5 min. The reaction was terminated by adding 2.5 μl of termination buffer supplemented with a 20-fold excess of unlabeled Oligo 8 to prevent spontaneous annealing during deproteination. The resulting products were resolved and imaged as described for the unwinding assay. Annealing activity was quantified by subtracting the signal of the no-protein control from that of the experimental samples.

### Electrophoretic mobility shift assay (EMSA)

The indicated amount of ZRANB3 was incubated with 10 nM DNA substrates in Buffer D (excluding 2 mM ATP) at 37°C for 20 min. Three microliters of loading buffer were then added, and the samples were analyzed on 2% TBE agarose gels, which were run in 1 × TBE buffer at 110 V for 40 min on ice. Fluorescence signals were subsequently analyzed as described for the unwinding assay.

### Footprinting analysis

Y30 DNA substrate (FAM-leading or FAM-lagging; 50 nM) was incubated with increasing concentrations of ZRANB3 (25, 50, 100, and 200 nM) in Buffer D at 37°C for 20 min to allow binding. Next, 50 mU of Benzonase (#E8263-5KU, Sigma) was added and incubated at room temperature for 3 min. The reaction was then deproteinated by adding 2.5 μl of termination buffer and incubating at 37°C for 15 min. An equal volume of 2 × denaturation dye (95% formamide, 0.1% Orange G, 10 mM Tris–HCl, pH 7.5, 1 mM EDTA, and 12% Ficoll PM400) was added, and the mixture was incubated at 95°C for 10 min to denature the DNA substrates. Samples were then analyzed on a 27% denaturing TBE–urea–PAGE (7 M urea) gel run in 1 × TBE buffer at 300 V for 180 min at 55°C. Gels were imaged using an Amersham™ Typhoon™ Biomolecular Imager (GE Healthcare) with a Cy2 525BP20 filter (515–535 nm). For quantification, the changes in band intensities between the 0 and 100 nM ZRANB3 samples were determined for the selected bands (labels 1–13 on the gel) and normalized to the intensities of the 0 nM ZRANB3 sample.

### Mass photometry

Mass photometry experiments were conducted using a TwoMP system (Refeyn Ltd., Oxford, UK). Glass coverslips coated with poly-lysine were thoroughly washed with pure water and 2-propanol, dried under a nitrogen stream, and subsequently used. ZRANB3 was diluted to 50 nM in Buffer D (excluding 0.1 μg/μl BSA) and then applied to a culture well gasket on the prepared coverslips. The protein-DNA complex was mixed well under the same conditions as above. Data acquisition and analysis were performed using AcquireMP and DiscoverMP software (Refeyn Ltd), with calibration conducted using a BSA standard of known molecular weight.

### Native MS

For native MS analysis, samples were buffer-exchanged into 150 mM ammonium acetate (pH 7.5) using Zeba Micro Spin Desalting Columns (40 kDa molecular weight cutoff (MWCO); Thermo Fisher Scientific) according to the manufacturer’s instructions. The samples were loaded into in-house gold-coated nano-ESI emitters and electrosprayed into a Q Exactive UHMR mass spectrometer (Thermo Fisher Scientific). The instrument parameters were as follows: spray voltage, 1.4 kV; capillary temperature, 200°C; S-lens RF level, 200; resolution, 6250. To optimize high-m/z ion transmission, the injection flatapole, inter-flatapole, bent-flatapole, and transfer multipole DC voltages were set to 5.0, 4.0, 2.0, and 0 V, respectively. To enhance desolvation, in-source collision-induced dissociation (CID) was set to 50 eV, higher-energy collisional dissociation (HCD) energy to 50 V (absolute), and in-source trapping to 50 V. The trapping gas pressure was set to 5.0. RAW files were analyzed manually using Thermo Xcalibur Qual Browser (version 2.0.3) and the peak intensities of mass species were quantified using UniDec (version 8.0.3). Measurement errors reflect the deviation of peak centroids for the same mass species in different charged states.

### Single-molecule fluorescence microscopy

#### Reaction chamber preparation

All single-molecule fluorescence assays were conducted on PEGylated slides and coverslips, as described previously [41]. The chamber was incubated with streptavidin (0.05 mg/ml, Thermo Fisher) for 3 min, followed by T50 buffer (20 mM Tris, 50 mM NaCl, pH 8.0) washing. Then, it was loaded with 20 pM biotinylated Cy3–Cy5 DNA substrates in T50 (∼400 molecules per field of view) for 3 min. DNA was immobilized via biotin–streptavidin linkage. Reactions were carried out in imaging buffer (35 mM Tris–HCl, 50 mM KCl, 2 mM MgCl_2_, 1 mM DTT, 1 mg/ml BSA, 8 mg/ml glucose, 2 mM Trolox, 2 mM COT, 60 U/ml glucose oxidase, 60 U/ml catalase, pH 7.5) to minimize photobleaching.

#### Image acquisition

The micro-mirror TIRF microscope was built based on a commercial framework (Mad City Labs Inc.) [42], with details described previously [43]. Images were collected through a 100× objective lens (UAPON 100XTIRF, Olympus) and split by a commercial dual-view system. The emission images passed through band-pass filters [FF01-517/20 (Semrock), FF01-572/15 (Semrock), FF01-676/29 (Semrock), ZET532/640m (Chroma) for the blue, green, red, and green + red channels, respectively] installed on a filter wheel (Lambda 10-B, Sutter). The images were acquired using an EMCCD system (Andor). The drift along the optical axis was corrected by tracking the position of the retro-reflected laser beam using a quadruple photo-diode. Image acquisitions were controlled by Glimpse, written in LabVIEW modified from Jeff Gelles' lab (https://github.com/gelles-brandeis/Glimpse).

#### ZRANB3 strand-displacement assay

In this series of experiments, Y15 DNA, ZRANB3, and the K163D mutant were used as the reactants. WT and K163D mutants of ZRANB3 were taken from a −80°C freezer, thawed at room temperature, and centrifuged before use. Then, 50 nM of the proteins were added to 20 μl imaging buffer in the presence of 2 mM ATP or AMPPNP. The reaction mixture was deoxygenated for 3 min at room temperature before being applied to the microfluidic chamber. Image acquisition was performed using sequential red–green–blue laser excitation (one frame per cycle), with 400 cycles corresponding to ∼16 min of recording. The exposure time was 0.5 s per frame.

Survival curves of the unwound fraction are based on the unwinding times of individual DNA molecules, with leading and lagging nascent strands scored independently. If nascent strands remained bound at the end of the observation timeframe, they were considered to be right-censored. Calculated unwinding times were then analyzed using the Kaplan–Meier estimator implemented in the Python lifelines package, yielding survival curves.

#### Fluorescence resonance energy transfer assay

A lagging-gap fork reversal substrate was used to investigate the fork reversal activity of ZRANB3. Before imaging, 0.5 nM ZRANB3^488^ was mixed with 2 mM ATP in imaging buffer. Imaging was performed using the green and blue lasers with a cycle consisting of two green frames followed by one blue frame for 200 cycles. The green laser’s emission filter set was green + red. The exposure time was 0.5 s per frame. In the final 10 frames, the red laser was switched on to confirm the presence of the Cy5 fluorophore.

#### ZRANB3 photobleaching and fork reversal assay

ZRANB3^488^ was diluted to 5 nM in imaging buffer in the absence of ATP and introduced into the microfluidic channel, followed by a 5-min incubation, allowing binding to DNA. After incubation, unbound ZRANB3^488^ was washed with imaging buffer containing 2 mM ATP. Before real-time recording, five snapshot frames were taken with the green laser and red laser to record the positions of the DNA substrates. Real-time imaging was then performed using high-intensity blue laser excitation: 500 frames in a single cycle, with a total recording time of ∼50 s. The exposure time was 0.1 s per frame. After photobleaching, fluorescence resonance energy transfer (FRET) imaging was performed using the green laser, with 30 frames per cycle and 20 cycles in total. The emission filter set used was green + red. The exposure time was 0.5 s per frame. In the final 10 frames, the red laser was switched on to confirm the presence of the Cy5 fluorophore. Successful fork reversal events were considered when FRET switched from a low-FRET state (DNA-only, <0.4) to a high-FRET state (>0.8) before the disappearance of both nascent strands. No Cy5 fluorescence intensity was observed in the final 10 frames of red laser excitation.

To analyze the fluorescence intensity of ZRANB3^488^ molecules, we defined “Areas of Interest (AOI)” of 5 × 5 pixels. The fluorescence intensity of a single AOI was defined as the difference between the pixel intensity sum within the AOI and the average pixel intensity of the surrounding region centered on the AOI. The final value was normalized by subtracting the background and dividing by the number of pixels within the AOI. The ZRANB3^488^ binding dwell time (τ_ZR) was determined as the time difference between binding and dissociation of ZRANB3^488^. Based on the calculated τ_ZR values, all traces were aligned, and the mean intensity of each frame was computed to obtain the mean ZRANB3^488^ signal intensity.

### Statistics

For biochemical assays (fork reversal, unwinding, annealing assays, EMSA, and DNA footprinting), data are presented as representative images, accompanied by bar plots showing the mean ± SEM. of at least three independent replicates. Each replicate corresponds to an independent protein preparation and DNA substrate preparation. No formal statistical hypothesis testing (*P*-values or effect size calculations) was performed, as these assays are descriptive and intended to demonstrate reproducibility and mechanistic trends. Shaded areas in single-molecule survival plots represent 95% confidence intervals.

## Results

### ZRANB3-catalyzed fork reversal relies on ATP hydrolysis and preferentially targets lagging-gap substrates

We expressed wild-type ZRANB3 and an ATPase-deficient mutant (K163D) in a human expression system and purified them to near homogeneity for downstream analyses (see Methods and Supplementary Fig. S1A). Fork reversal assays were conducted using fluorescently-labeled substrates bearing a 30-nucleotide (nt) gap on either the leading or lagging strand (Supplementary Fig. S1B and C, panel i). In these assays, ZRANB3-mediated fork reversal generated parental and nascent duplex DNA that could be resolved by native polyacrylamide gel electrophoresis for visualization. Consistent with previous finding [23, 24, 31], we found that ZRANB3 catalyzed fork reversal on both types of substrates in a concentration-dependent manner. Importantly, no reaction product was generated by the ATPase-deficient K163D mutant (Supplementary Fig. S1B and C), confirming that fork reversal requires ATP hydrolysis [31]. Moreover, our results indicate that ZRANB3 acted more efficiently on the substrate with a DNA gap in the lagging strand (Supplementary Fig. S1D).

### ZRANB3 possesses intrinsic DNA unwinding activity

ZRANB3 has not been demonstrated previously as having DNA unwinding activity by applying the partial duplex DNA substrate [35]. Given that reversal initiates at the fork structure, we designed a fork-structured DNA substrate, Y15, to determine whether ZRANB3 possesses unwinding activity to separate the nascent strand DNA. Y15 comprises two 15-nt nascent strands (Fig. 1B-i) with heterologous sequences on the arm to prevent the annealing of the parental strands. Incubation of ZRANB3 with Y15 led to clear unwinding of both duplex regions in the fork arms in both a protein concentration- and time-dependent manner (Fig. 1B and C). Notably, the duplex harboring the leading strand was unwound more efficiently than that with the lagging strand (Fig. 1B and C). Further experiments using a substrate with the identical sequence on the two splayed arms provided evidence that this preference is not sequence-dependent (Supplementary Fig. S2A). Importantly, duplex unwinding required ATP hydrolysis as no DNA unwinding occurred in the presence of ADP or nonhydrolyzable ATP analogs (Fig. 1D), and the ATPase-deficient ZRANB3 K163D mutant lacked unwinding activity (Fig. 1E).

**Figure 1.**
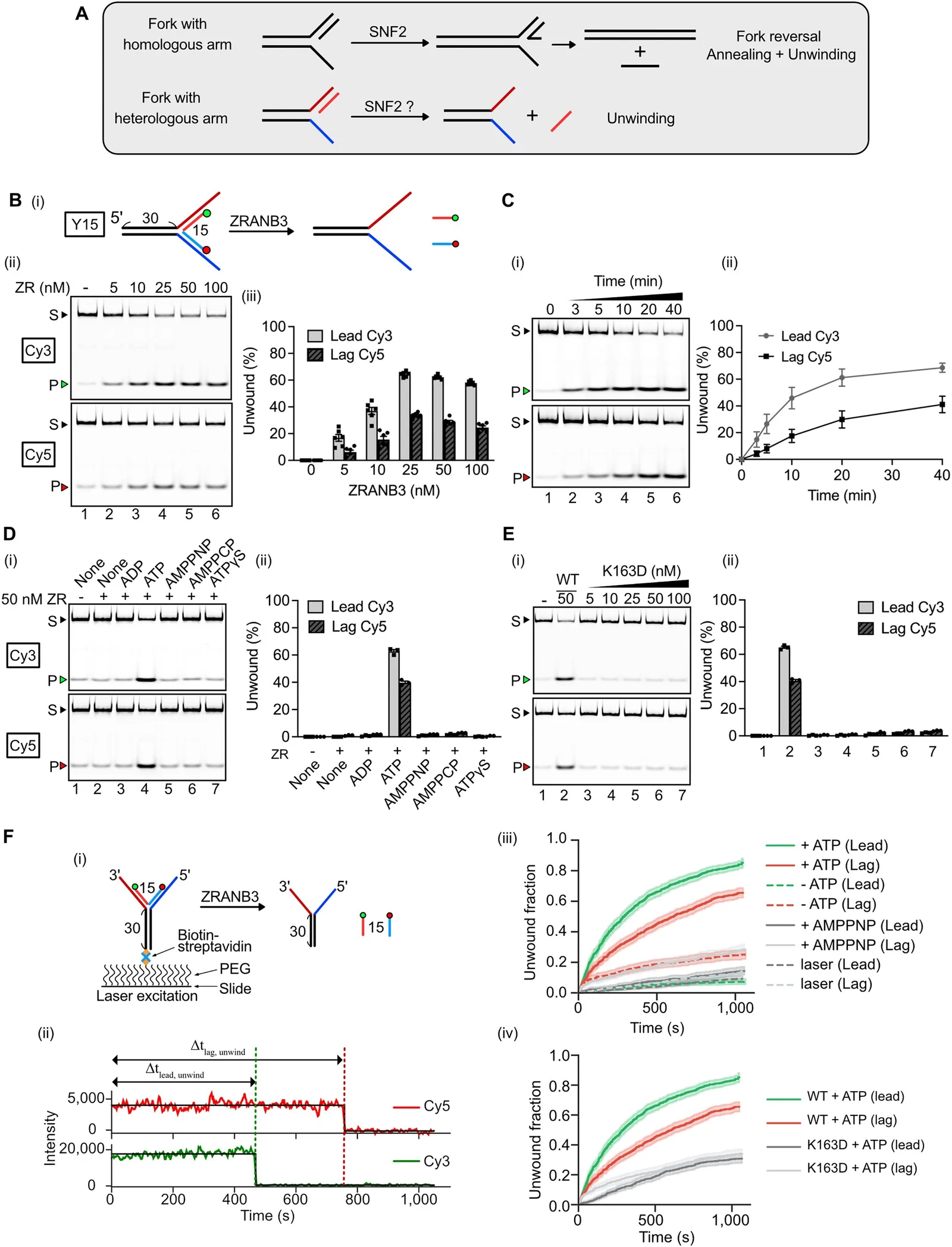
ZRANB3 exhibits ATP–dependent unwinding activity. (**A**) Schematic of unwinding on homologous versus heterologous arms. Top: On forks with homologous arms, SNF2 translocases drive fork reversal via annealing and unwinding. Bottom: On forks with heterologous arms (red and blue), parental re-annealing is blocked. Whether SNF2 translocases possess the intrinsic unwinding activity to separate nascent strands in the absence of annealing remains unclear. (**B**) Unwinding of a model fork substrate (Y15) by ZRANB3. (i) Schematic of Y15: red and blue lines mark noncomplementary splayed–arm sequences; green and red dots are Cy3 and Cy5 labels. (ii) Titration of ZRANB3 on Y15 (10 nM); products were resolved by native polyacrylamide gel electrophoresis (PAGE) and detected by fluorescence imaging. (iii) Quantification of the unwound fraction [*n* = 6; mean ± standard error of the mean (SEM)]. (**C**) Time-course of fork unwinding. ZRANB3 (50 nM) was incubated with Y15 for the indicated times before quantifying unwound products (*n* = 5; mean ± SEM). (**D, E**) ATP hydrolysis is required for duplex unwinding. Unwinding assays were performed with wild–type ZRANB3 in the presence of various ATP analogs (**D**) or with the ATPase–dead mutant K163D in the presence of ATP (**E**). (i) Representative gels. (ii) Quantification of unwound products (*n* = 3; mean ± SEM). (**F**) Single–molecule fluorescence analysis of ZRANB3 unwinding activity. (i) Biotin–Y15 tethered to a PEG–coated coverslip; Cy3 (green)-labeled leading and Cy5 (red)-labeled lagging nascent strands were used to track their displacement. (ii) Representative fluorescence traces: a single-step drop in Cy3 or Cy5 signal indicates strand displacement. ∆t_unwind_ is defined as the interval from ZRANB3 addition to loss of fluorescence signal. (iii, iv) Unwound fractions derived from ∆t_unwind_ for wild–type ZRANB3 with AMPPNP (iii) and for the K163D mutant with ATP (iv). Dashed line laser–only controls account for photobleaching and spontaneous dissociation.

In parallel, we employed single-molecule fluorescence colocalization microscopy to observe ZRANB3-mediated unwinding events directly in real-time. For these assays, the Y15 substrate was immobilized on a PEGylated surface via biotin-streptavidin interaction (Fig. 1F-i). Fluorescently-labeled DNA was illuminated using rapidly alternating laser excitation in the presence of ZRANB3. Nascent strand displacement was detected by a one-step drop in fluorescence signal intensity (Fig. 1F-ii). Kinetic analyses to measure dwell time (Δt_unwind_) revealed that, consistent with results from our bulk biochemical assays, leading strands were displaced more rapidly and efficiently compared to lagging strands (Fig. 1F-iii). This strand displacement preference persisted even when fluorescent dyes were swapped for nascent strands (Supplementary Fig. S2B), ruling out dye photostability issues. Control assays confirmed the ATP dependency of strand displacement and that photobleaching had a minimal impact on our observations (Fig. 1F-iii and F-iv). Collectively, these results from both bulk and single-molecule analyses demonstrate that ZRANB3 exhibits intrinsic unwinding activity that can efficiently unwind duplex DNA in an ATP-dependent manner in the context of a replication fork structure.

### ZRANB3 preferentially initiates DNA unwinding at a fork junction

To investigate how DNA structure impacts ZRANB3’s unwinding efficiency, we compared fork substrates (Y15 leading-blunt and Y15 lagging-blunt) with substrates featuring 5′ or 3′ overhang structures (Fig. 2A). Our results clearly demonstrate that ZRANB3 preferentially unwinds fork-like substrates (Fig. 2A-i and A-ii). However, ZRANB3 associates with both fork and overhang substrates in the absence of ATP (Fig. 2B), indicating that its substrate selectivity for DNA unwinding cannot be attributed simply to a lack of DNA association but instead reflects a requirement for specific structural features of the fork junction. Given that ZRANB3 preferentially unwinds forked DNA structures, we postulated that the fork junction might be crucial for initiating nascent strand displacement. To test this hypothesis, we assessed if the distance between the nascent strand DNA and the fork junction influences ZRANB3’s unwinding efficiency. To do so, we constructed leading-gap substrates with no gap (Y15), a 5-nt gap (Lead gap 5), or a 10-nt gap (Lead gap 10) adjacent to the fork junction (Fig. 2C-i). Since ZRANB3 displays structure-specific endonuclease activity to cleave gap substrates near the branch point [31–33], we conducted our analysis using the nuclease-deficient H1021A variant [32] (Supplementary Fig. S3A). As expected, endonuclease activity was abolished in the H1021A mutant (Supplementary Fig. S3B), yet it retained the DNA unwinding activity of wild-type enzyme (Supplementary Fig. S3C). Our experiments revealed a substantial reduction in leading-strand unwinding activity when a 5- or 10-nt gap was introduced on the leading arm compared to the substrate with no such gap (Fig. 2C-ii and C-iii). In contrast, the absence of such a gap did not significantly impact lagging-strand unwinding since the lagging-strand is accessible for ZRANB3 from the junction (Fig. 2C-ii and C-iv). Collectively, these findings strongly support that ZRANB3 initiates DNA unwinding specifically at the fork junction.

**Figure 2.**
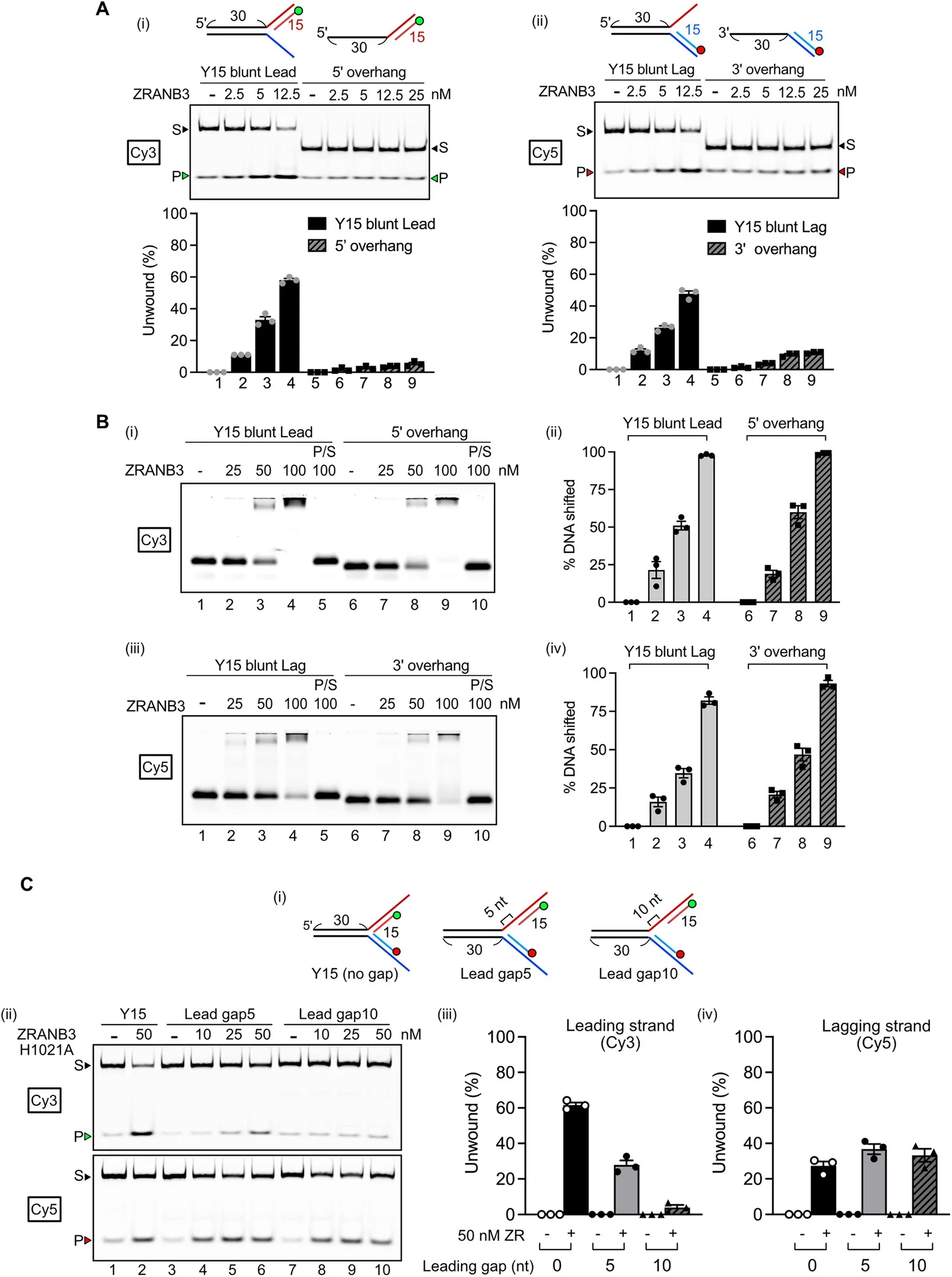
Fork–junction proximity governs ZRANB3 unwinding activity. (**A**) Unwinding efficiency on fork–like DNA substrates (10 nM). Comparison of Y15 blunt-lead/5′–overhang (i) and Y15 blunt-lag/3′–overhang (ii) substrates (*n* = 3; mean ± SEM). (**B**) DNA–binding of ZRANB3. Titration of ZRANB3 on Y15 blunt-lead/5′–overhang (i) or blunt-lag/3′–overhang (iii) substrates (10 nM). (ii, iv) Quantification of the shifted DNA in (i) and (iii), respectively (*n* = 3; mean ± SEM). Reaction mixtures were treated with SDS and proteinase K (P/S) in lanes 5 and 10 to deproteinize the nucleoprotein complex as a control. (**C**) Effect of nascent strand–junction distance on the activity of the nuclease–dead ZRANB3 H1021A variant. (i) Schematics of the Y15 (0-nt gap), Lead gap 5, and Lead gap 10 substrates; Cy3 (green) and Cy5 (red) mark leading and lagging strands. (ii) Dose–dependent unwinding assays with 10 nM of indicated DNA substrates. (iii, iv) Quantification of unwound leading (iii) and lagging (iv) strands (*n* = 3; mean ± SEM).

### ZRANB3 binds fork DNA at the junction in a predominantly 1:1 stoichiometry

Our results have demonstrated that duplex unwinding by ZRANB3 requires proximity of the nascent strand to the fork junction. We performed DNA footprinting analysis to help delineate the unwinding mechanism and to map the position of ZRANB3 occupancy on the fork substrate. In these assays, Y30 DNA substrates fluorescently labeled with FAM on either the leading or lagging strand were incubated with purified ZRANB3, before performing Benzonase treatment (see Supplementary Fig. S4A for schematic). Digested products were then resolved on denaturing gels and visualized by fluorescent imaging. The DNA substrate evidently became better protected as ZRANB3 concentration increased (Supplementary Fig. S4B). We selected degraded DNA bands at random across the entire Y30 substrate and quantified their signal intensities. Plot analysis to compare the percentage digested of each band with the control clearly revealed that ZRANB3 protected the parental strands proximal to the fork junction (Fig. 3A, compare bands 4–6 on the leading strand and lanes 5–7 on the lagging strand with other lanes). This footprint aligns closely with our unwinding analyses, providing further evidence that duplex unwinding initiates at the fork junction.

**Figure 3.**
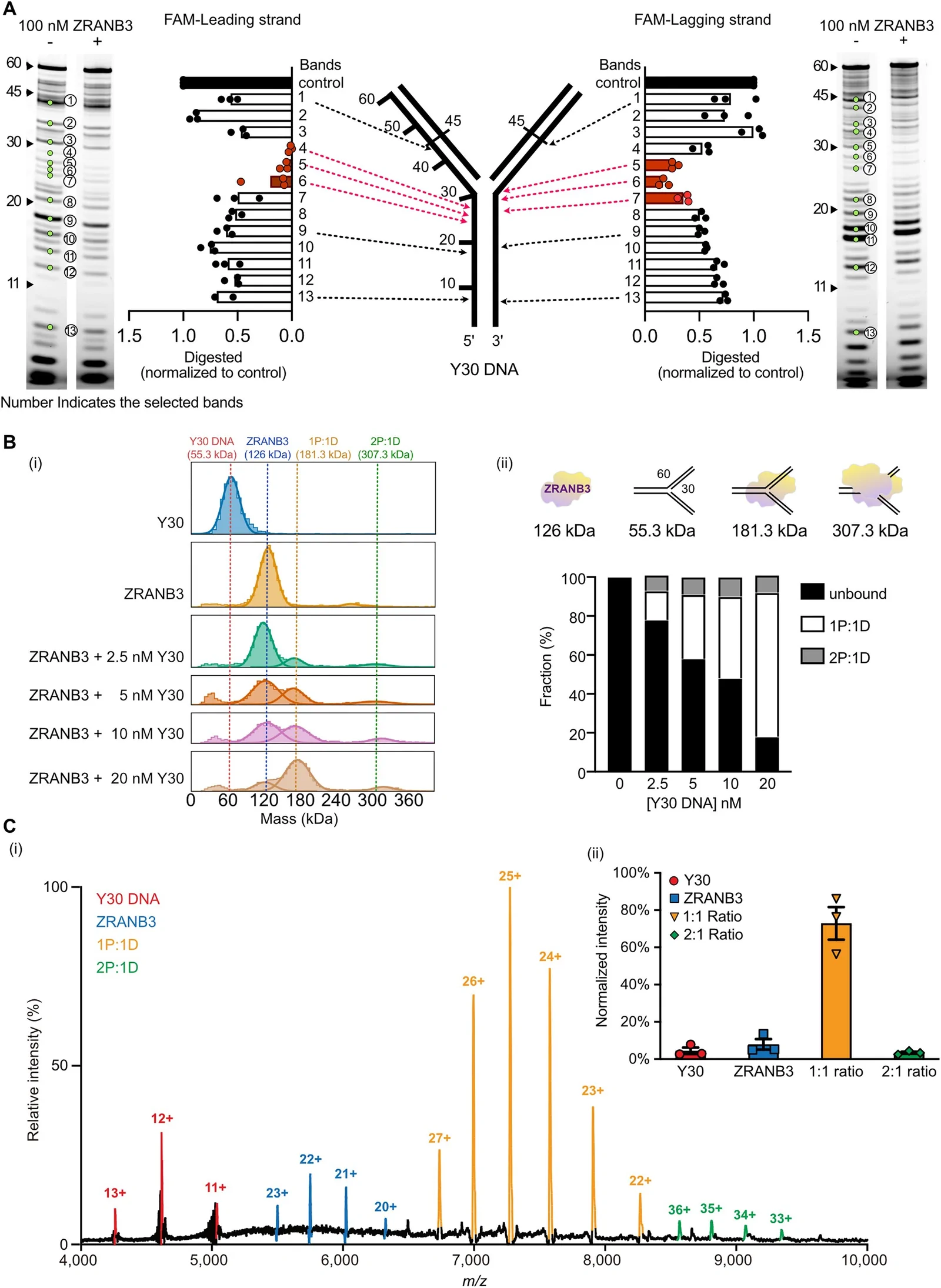
ZRANB3 binds the fork junction predominantly as a monomer. (**A**) DNA footprinting analysis on a 60–bp fork substrate (Y30, 50 nM) labeled with FAM on either the leading or lagging strand (schematic in Supplementary Fig. S4). Protections were mapped by nuclease digestion—band intensities in the presence of ZRANB3 were normalized to no–protein controls to yield the protection ratios (*n* = 3; mean ± SEM). The most strongly protected regions on both arms of the junction are indicated by the red bars. (**B**) Mass photometry of ZRANB3-Y30 complexes. (i) Representative mass-distribution peaks for 50 nM ZRANB3 with 0, 2.5, 5, 10, or 20 nM Y30. (ii) Quantification of the ZRANB3:DNA stoichiometry, showing the predominance of the 1:1 complex. (**C**) Native MS characterization of ZRANB3 (10 µM) co-incubated with Y30 (5 µM). (i) The representative spectrum identifies species corresponding to free Y30 (red), the ZRANB3 monomer (blue), the 1:1 complex (orange), and the 2:1 complex (green). (ii) Quantitative analysis of the relative abundances of each identified species (*n* = 3).

To further elucidate the unwinding mechanism, next we determined the binding stoichiometry of ZRANB3 to the fork substrate by means of mass photometry. Our analysis showed that ZRANB3 is predominantly monomeric (126 kDa), with a small fraction forming dimers, which is consistent with a previous report [44]. Increasing concentrations of the Y30 substrate (55.3 kDa) induced a peak shift from ∼126 kDa to 181 kDa, indicating formation of a 1:1 protein-DNA complex (Fig. 3B and Supplementary Fig. S5). Quantitative analysis further revealed that the majority of complexes consisted of monomeric ZRANB3 bound to Y30, with only a minor proportion (∼10%) exhibiting a 2:1 stoichiometry (Fig. 3B-ii). Considering that the stoichiometry of ZRANB3 and its complex with the DNA fork may be influenced by protein concentration, we further employed high-resolution native MS at elevated ZRANB3 concentrations (µM range) to more accurately determine the binding stoichiometry. With improved resolution, native MS identified distinct mass species corresponding to Y30 DNA, ZRANB3, the 1:1 protein–DNA complex, and the 2:1 protein–DNA complex, each exhibiting < 1% deviation compared to their theoretical masses (Supplementary Table S3). These results enabled accurate determination of ZRANB3 complex stoichiometry, with no evidence of alternative binding arrangements (Fig. 3C). Consistent with mass photometry data, quantitative analysis of the MS spectra revealed a predominant 1:1 complex (∼70%), together with a minor population of 2:1 complex (3.5%, Fig. 3C-ii). Collectively, these findings indicate that ZRANB3 primarily binds to the fork junction in a 1:1 ratio.

### ZRANB3 initiates long-range fork reversal via coupled unwinding and strand annealing activities

It has been documented previously that the average length of the regressed arm of a replication fork observed in human cells is ∼300 bp [9]. Consistent with this physiological scale, the plasmid-based fork reversal assay has demonstrated that ZRANB3 efficiently catalyzes long-range fork reversal [24], further highlighting its robust remodeling capacity. Our current study has demonstrated that ZRANB3 possesses intrinsic unwinding activity, prompting the question of its processivity in duplex separation. To address this topic, we evaluated fork DNA substrates with nascent strand lengths of 15, 20, and 30 bp for their unwinding efficiency (Fig. 4A). Surprisingly, we observed minimal unwinding activity on substrates with nascent strands longer than 20 bp, implying limited processivity of ZRANB3-mediated unwinding activity. Interestingly, despite this low processivity, ZRANB3 efficiently catalyzed fork reversal for substrates containing 52-bp splayed-arm duplex DNA (Supplementary Fig. S1B–D). Therefore, another biochemical attribute of ZRANB3 likely contributes to its ability to promote extended fork reversal. It has been documented previously that ZRANB3 also possesses ssDNA annealing activity [44]. Consistent with that report, we found that ZRANB3 displays robust single-strand annealing activity (Fig. 4B). Importantly, this annealing activity occurs independently of ATP hydrolysis (Fig. 4B, compare lanes 2–5 and 7–10). Collectively, our results and the previously published study [44] highlight that ZRANB3 exerts two distinct activities, namely, ATP hydrolysis-dependent duplex unwinding and ATP-independent DNA strand annealing. We hypothesize that localized duplex unwinding by ZRANB3 facilitates annealing of the displaced parental strands, thereby promoting the initial formation of a four-way junction during fork reversal (Fig. 4C).

**Figure 4.**
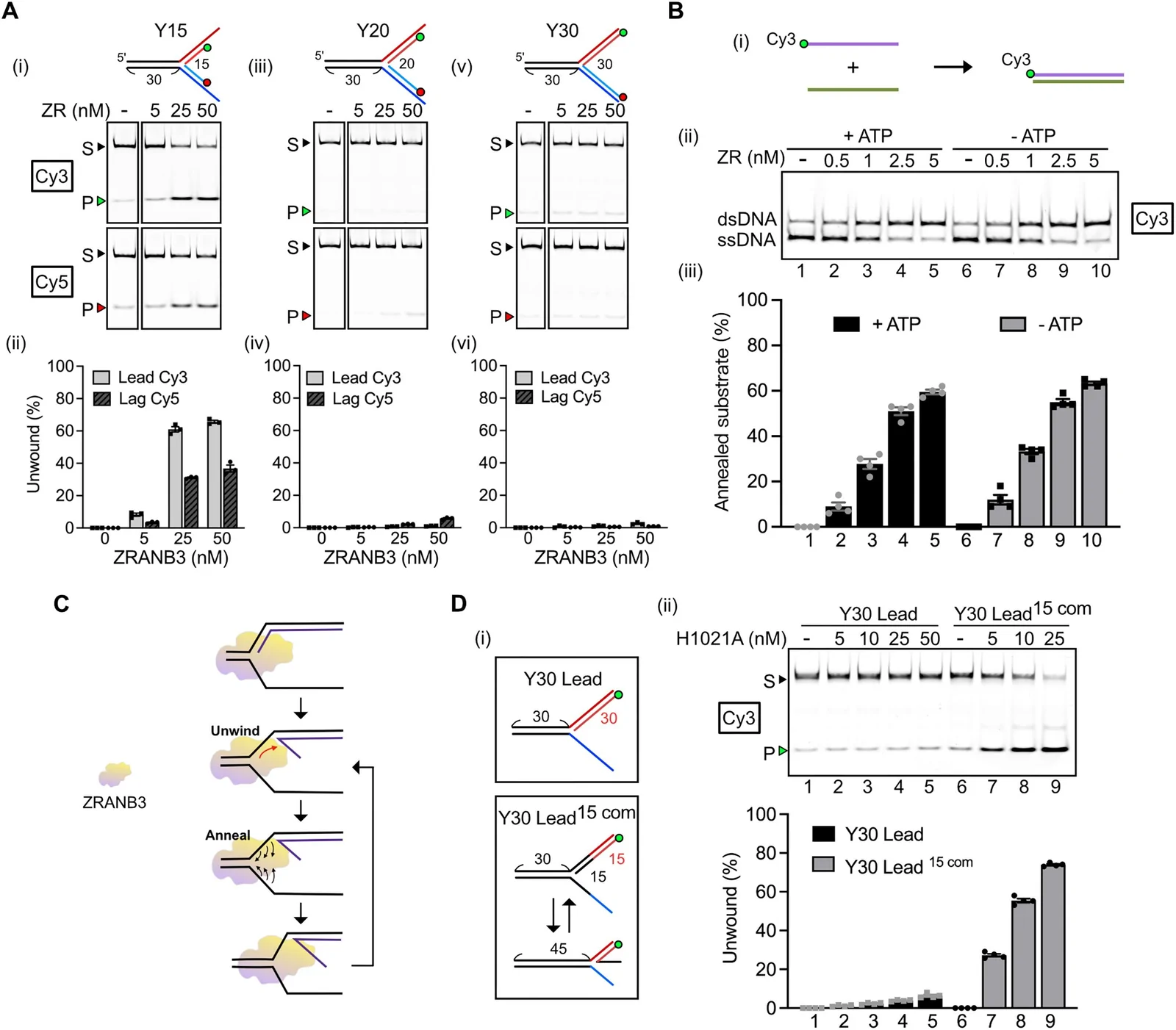
Unwinding and strand annealing activities of ZRANB3 facilitate replication fork reversal. (**A**) Processivity of ZRANB3 unwinding. Fork substrates bearing 15–, 20–, or 30–bp nascent strands (10 nM each) were incubated with the indicated amount of ZRANB3. Unwound products were then resolved and quantified (*n* = 3; mean ± SEM). (**B**) ATP–independent ssDNA annealing by ZRANB3. (i) Two complementary oligonucleotides (3 nM each) were incubated with ZRANB3 in the presence or absence of ATP, and then the annealed duplexes were analyzed (ii) and quantified (iii) (*n* = 4; mean ± SEM). (**C**) Schematic model in which localized unwinding and parental-strand annealing facilitate the initial remodeling of the replication fork. (**D**) Parental-strand annealing facilitates extended ZRANB3-mediated unwinding. (i) Comparison of unwinding on Y30–Lead (30 nt noncomplementary) versus Y30–Lead^15 com^ (first 15-nt adjacent to the junction complementary to the parental strands) substrates. Nuclease–dead ZRANB3 (H1021A) was used to avoid substrate cleavage. Red/blue, noncomplementary; black, complementary. (ii) Representative gel images and quantification of unwound products (*n* = 4; mean ± SEM).

To test this model further, we performed unwinding assays using a Y30 Lead^15 com^ substrate, which possesses a 30-bp leading arm in which the first 15 nt are complementary to the parental strands, permitting junction migration over this 15-bp window (Fig. 4D-i). The nuclease-dead ZRANB3 H1021A variant was used in this experiment to avoid confounding DNA cleavage. A Y30 Lead substrate, which lacks complementary sequence, served as a negative control. As expected, only trace levels of unwinding products were generated from the control substrate, reflecting limited unwinding processivity and no annealing region (Fig. 4D-ii, lanes 2–5). In contrast, the Y30 Lead^15 com^ substrate supported robust ATP-dependent unwinding of the full 30-bp duplex by ZRANB3 (Fig. 4D-ii, lanes 7–9; and Supplementary Fig. S6).

### Monomeric ZRANB3 catalyzes successful fork reversal

To determine if monomeric ZRANB3 is capable of catalyzing fork reversal, we subjected fluorescently-labeled ZRANB3 to a single-molecule FRET-based assay. SNAP-tagged ZRANB3 was purified and conjugated with Alexa Fluor 488 (designated ZRANB3^488^; Supplementary Fig. S7A). Importantly, ZRANB3^488^ retained a level of fork reversal activity comparable to that of an unmodified counterpart (Supplementary Fig. S7B). In the first set of experiments (Fig. 5A-i), a fork reversal substrate containing a 30-nt lagging gap was immobilized on a surface, with the Cy5 (red) tag positioned on the leading strand and the Cy3 (green) tag on the lagging strand. After introducing ZRANB3^488^ and washing away excess protein, we determined the number of bound ZRANB3^488^ molecules by single-molecule photobleaching analysis upon 488-nm excitation (Ex488, blue bar), while fork reversal was tracked through changes in FRET between the Cy3/Cy5 pair. Approximately 80% of successful fork reversal events exhibited single-step photobleaching, consistent with a single ZRANB3^488^ molecule bound to the DNA substrate. A representative Alexa488 trace showed a single photobleaching step (Fig. 5A-ii, top), indicating substrate engagement by monomeric ZRANB3. Concurrently, changes in Cy3 and Cy5 signal intensity (Fig. 5A-ii, middle) were consistent with a low-to-high FRET transition (Fig. 5A-ii, bottom), as expected for initiation of fork reversal. Completion of fork reversal was marked by co-disappearance of the Cy3 and Cy5 signals. These results demonstrate that pre-bound monomeric ZRANB3 is capable of driving fork reversal.

**Figure 5.**
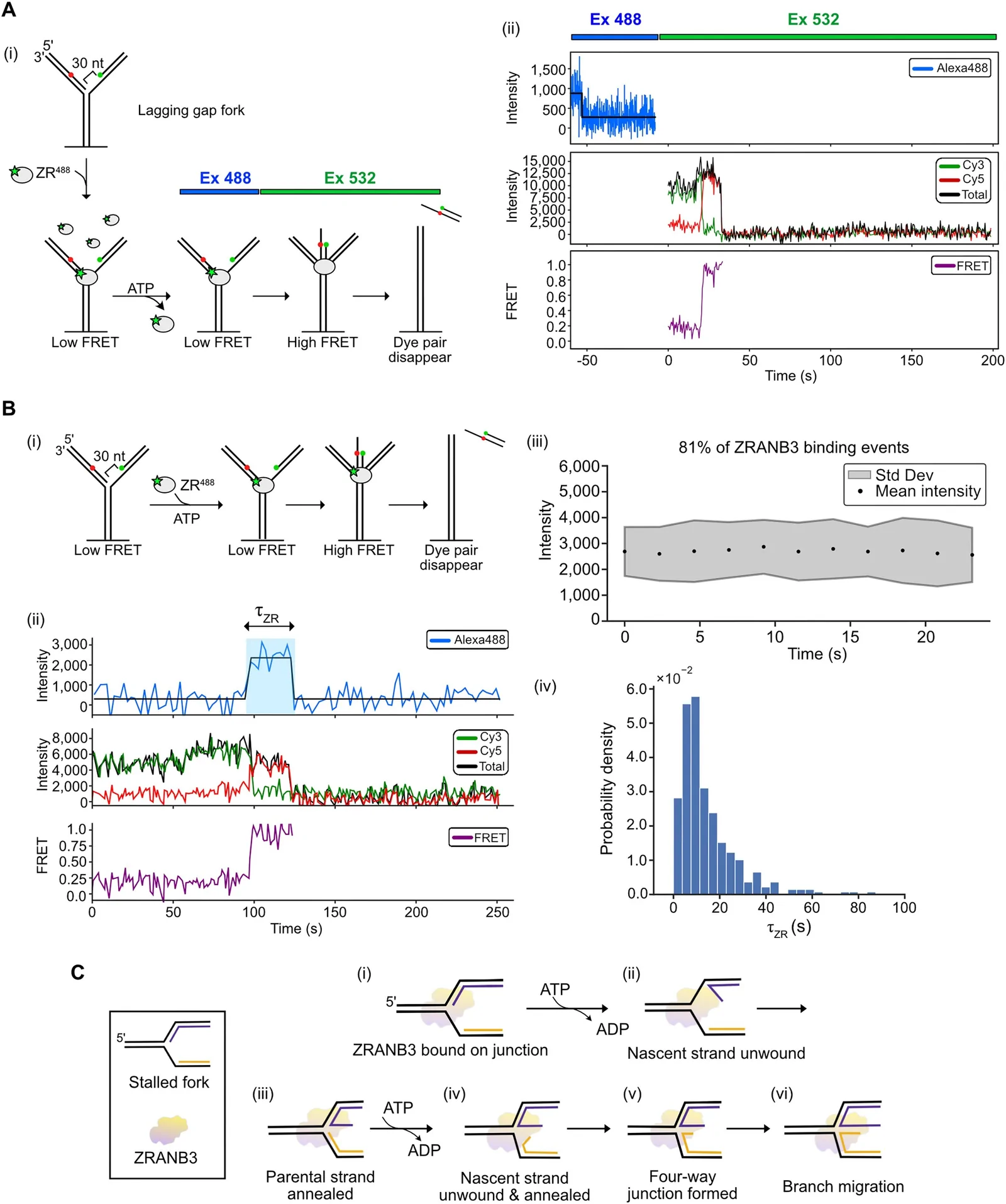
Monomeric ZRANB3 is able to catalyze fork reversal. (**A**) The monomeric state of pre-bound ZRANB3 can successfully promote fork reversal. (i) Schematic of the single-molecule photobleaching and FRET experimental design. ZRANB3^488^ was preincubated and washed out with imaging buffer containing ATP. Photobleaching of ZRANB3^488^ (blue bar, Ex488) returns the monomeric protein state and a change in FRET validates active fork reversal events (green bar, Ex 532). (ii) Representative fluorescence traces of Cy5 (red), Cy3 (green), and Alexa488 (blue). A single photobleaching step of pre-bound ZRANB3^488^ (black, top panel) is followed by a transition from low to high FRET efficiency (purple, bottom panel), and a single drop in total intensity (black, middle panel) indicates a successful fork reversal event. (**B**) Real-time successful fork reversal occurs during a ZRANB3 binding event without sequential ZRANB3 binding or dissociation. (i) Schematic of the single-molecule experiment to observe ZRANB3 binding and fork reversal in real-time. (ii) Representative fluorescence traces corresponding to ZRANB3^488^ binding (top panel, blue trace) and the fork reversal process indicated by FRET (middle and bottom panels). The blue-shaded region denotes the ZRANB3 binding dwell time, defined as τZR. (iii) Time-binned average fluorescence intensity of all detected ZRANB3^488^ binding events leading to successful fork reversal (*n* = 166). The experiments were repeated three times with similar results. (iv) Histogram of ZRANB3^488^ binding dwell times (τZR) leading to successful fork reversal from three independent experiments (*n* = 364, *N* = 3). (**C**) Proposed mechanism of ZRANB3-mediated fork reversal: (i) ZRANB3 binds the fork junction as a monomer; (ii) ATP-dependent localized duplex opening disrupts the leading nascent strand adjacent to the junction; (iii) the exposed parental strands anneal; (iv) subsequent ATP-dependent unwinding of the lagging nascent strand facilitates further strand rearrangement; (v) reciprocal strand annealing completes formation of the four-way junction; and (vi) ZRANB3 promotes branch migration to drive long-range fork reversal.

We also designed a second set of single-molecule experiments to monitor in real-time ZRANB3^488^ binding and the FRET transitions associated with fork reversal (Fig. 5B-i). A representative trace showed that ZRANB3^488^ binding coincided with a shift from the low-FRET to the high-FRET state, followed by loss of Cy3, Cy5, and Alexa488 signals (Fig. 5B-ii). To evaluate ZRANB3 occupancy, we quantified Alexa488 signal intensity during individual active reversal events. The binding period, as indicated by blue shading, showed a stable average intensity (Fig. 5B-iii), confirming that no additional ZRANB3^488^ molecules were recruited. Dwell-time analysis (τ_ZR_) revealed that most binding events (81%) lasted <23 s, consistent with the timescale of observed FRET transitions (Fig. 5B-iv). Together, these data help establish that the monomeric ZRANB3 molecule is capable of successfully catalyzing fork reversal.

## Discussion

Our study unveils the mechanism by which ZRANB3 mediates replication fork reversal. Specifically, we show that ZRANB3 exhibits limited DNA unwinding activity on a fork substrate with heterologous arms. Our detailed mechanistic analysis of ZRANB3 reveals that it engages the DNA fork junction as a 1:1 nucleoprotein complex, and that its unwinding activity, though limited in processivity, acts locally at the fork junction to promote the initiation of fork regression. Our biochemical analyses identify two activities of ZRANB3 relevant to fork remodeling: ATP-hydrolysis-driven localized duplex opening and ATP-independent ssDNA annealing. We propose that these activities facilitate the initial remodeling of the replication fork toward formation of a four-way junction. Moreover, the findings from our single-molecule FRET-based assays demonstrate that a single ZRANB3 molecule is sufficient to efficiently catalyze fork reversal. Although our complementary assays independently demonstrate ZRANB3-mediated duplex unwinding, strand annealing, and fork reversal, our current single-molecule configuration does not permit simultaneous visualization of duplex opening and parental-strand annealing within the same fork reversal event. Future development of multiplexed single-molecule approaches should allow the proposed temporal coordination of these activities to be directly examined.

We note that previous *in cellulo* analysis of the ZRANB3 K163D mutant variant, which is defective in ATP hydrolysis, has provided compelling evidence that the biological activity of ZRANB3 in replication fork reversal requires its ATPase activity [20, 21]. Based on our findings and those of previously published studies [20–26, 31], we propose a model whereby localized duplex unwinding and strand annealing facilitate the critical initial transition from a three-way replication fork to a four-way junction (Fig. 5C). In this model, ZRANB3 initiates fork reversal at the fork junction by locally disrupting the nascent duplex through ATP hydrolysis. We propose that the resulting local strand dynamics facilitate annealing of the parental strands, thereby promoting formation of the four-way junction. This model is further supported by our experiments using a fork substrate containing a 15-nt mobile region, which allows annealing of the parental arm to facilitate the unwinding of downstream dsDNA regions (Fig. 4D and Supplementary Fig. S6). Once this four-way regressed fork is established, ZRANB3 remains continuously engaged at the moving junction to drive long-range reversal. Because branch migration is inherently isoenergetic, subsequent progression does not necessarily require repeated cycles of duplex opening and annealing or the formation of discrete unwound intermediates. Instead, local base-pair disruption and reciprocal base-pair formation are inherently coupled at the moving junction. We propose that ATP-dependent ZRANB3 translocation provides the energy and directionality required for this otherwise bidirectional branch migration, thereby promoting long-range fork reversal.

A model of concerted unwinding and annealing has been proposed, where strand displacement is observed in conjunction with homologous re-annealing [22, 23, 25]. For instance, magnetic tweezer assays using DNA hairpins demonstrated that SMARCAL1-mediated oligonucleotide displacement is driven by the re-annealing of the hairpin substrate [23]. In the current study, we employed a fork DNA substrate with heterologous splayed arms to specifically abolish the annealing capacity of the translocases. Under these conditions, we nonetheless observed the active displacement of 15-nt nascent strands. Supportively, the crystal structure of the HLTF HIRAN domain in complex with a 14-bp duplex revealed that binding induces partial unwinding of three nucleobases [45]. This structural precedent provides a basis for the protein’s intrinsic capacity to destabilize the nascent-parental duplex, facilitating the separation of the leading nascent strand. Our data demonstrate that active, ATP-dependent strand separation is an intrinsic and mechanistically essential component of fork reversal.

The prokaryotic DNA helicase RecG serves as a mechanistic paradigm for replication fork regression, utilizing a specialized wedge domain to coordinate duplex unwinding and reannealing into a four-way junction [46]. Structural and biochemical studies have established that RecG operates as a functional monomer to promote simultaneous strand separation [37, 40]. While mass photometry confirms that human SNF2 translocases are primarily monomeric in solution [44], their functional stoichiometry upon DNA engagement appears to be protein-specific. For instance, EMSA analysis of SMARCAL1 revealed multiple nucleoprotein species, suggesting the binding of >1 molecule to DNA [22]. Furthermore, recent native MS data indicated that HLTF forms functional dimers upon ATP addition to remodel the fork [47]. In contrast to these diverse assembly modes, our mass photometry and native MS data indicated that ZRANB3 bound to the fork DNA as a monomer in the presence of ATP. Moreover, our single-molecule experiments demonstrate that a solitary ZRANB3 monomer is sufficient to catalyze the entire fork reversal process. This distinction highlights a mechanistic plasticity within the SNF2 family. Rather than a singular universal model, these translocases appear to utilize tailored assembly strategies ranging from the streamlined monomeric efficiency of ZRANB3 to the potential multi-molecular engagement of HLTF and SMARCAL1 to fine-tune their roles in genome maintenance.

## Supplementary Material

gkag1034_Supplemental_File

## Data Availability

The data underlying this article are available in the article and in its online Supplementary Material.
