# Supplementary material for: Monomeric ZRANB3 drives replication fork reversal
Source: Nucleic Acids Res. 2026 Nov 3;54(20):gkag1034. doi: 10.1093/nar/gkag1034 (PMC13728640; doi:10.1093/nar/gkag1034)
Supplement: Supplementary file 1 [file gkag1034_supplemental_file.pdf]

Supplementary Information

Monomeric ZRANB3 drives replication fork reversal

Yen-Ju Chen[1], Yu-Hui Chiu[1], Chieh-Kai Liu[2], Hsuan Hsu[2], Kuan-Chi Wang[2], Guan-Ting Lian[1,3,4], Yi-An Chen[3], Sheng-Wei Lin[3], Hsin-Yung Yen[3], Hung-Wen Li[2]* & Peter Chi[1,3]*

[1] Institute of Biochemical Sciences, National Taiwan University, Taipei, Taiwan.

[2] Department of Chemistry, National Taiwan University, Taipei, Taiwan

[3] Institute of Biological Chemistry, Academia Sinica, Taipei, Taiwan

[4] Chemical Biology and Molecular Biophysics Program, Taiwan International Graduate Program, Academia Sinica, Taipei, Taiwan

*Corresponding author. Email: hwli@ntu.edu.tw, peterhchi@ntu.edu.tw

Supplementary Figures S1–S7
Supplementary Tables S1–S3

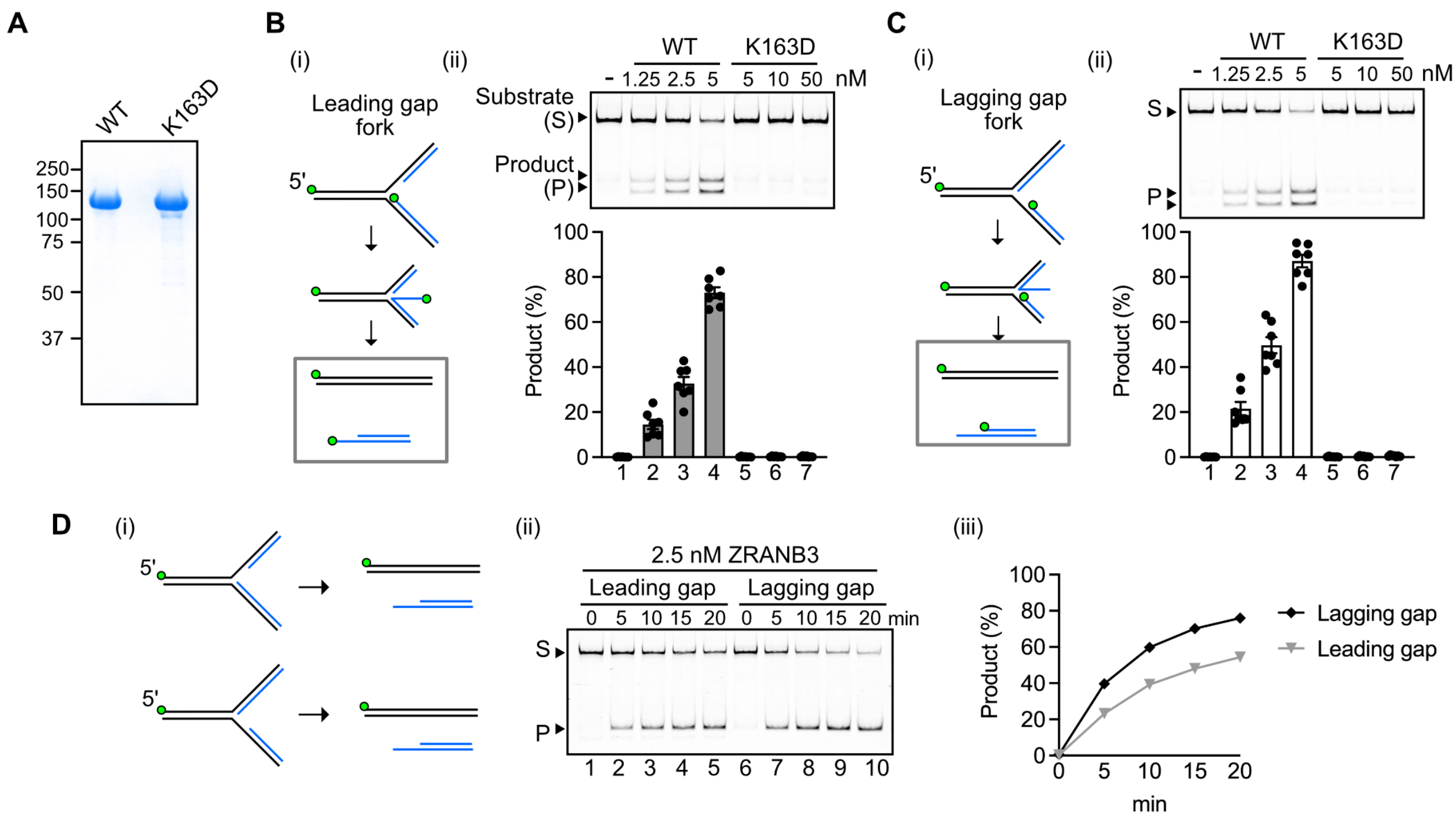

Supplementary Figure S1. Functional characterization of recombinant ZRANB3.

(A) SDS–PAGE analysis of purified wild-type (WT) and ATPase-dead mutant (K163D) ZRANB3 proteins. Proteins were resolved by 10% SDS–PAGE and stained with Coomassie blue. **(B,C)** Fork-reversal activity of ZRANB3 on leading-gap (B) and lagging-gap (C) substrates (16 nM). (i) Schematic representation of the fork-reversal assay substrates. (ii) Titration of WT or K163D ZRANB3 on DNA substrates. Fork reversal products were resolved by native PAGE and quantified (n = 7; mean ± s.e.m.). **(D)** ZRANB3 preferentially catalyzes fork reversal with lagging-gap substrates (16 nM). (i) Experimental schematic comparing leading- and lagging-gap substrates. DNA substrates (16 nM) were incubated with ZRANB3 at 37 °C for the indicated times. (ii) Representative gels and (iii) quantification of fork reversal products (n = 3; mean ± s.e.m.). In B–D, green dots indicate the Cy3 fluorescent label at the 5’ end of the specified oligonucleotide.

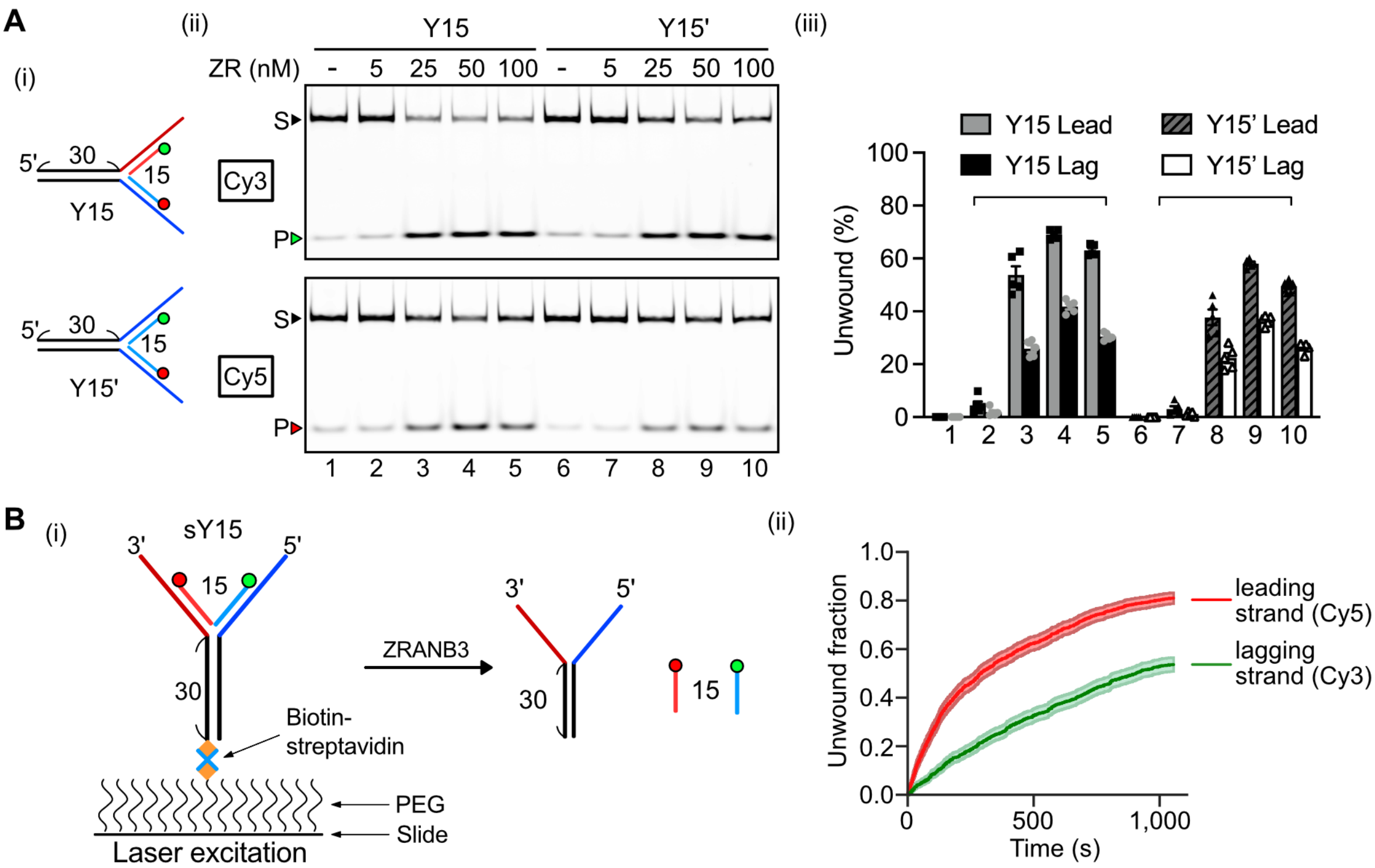

Supplementary Figure S2. The ZRANB3 preference for leading-strand unwinding is independent of DNA sequence and fluorescent dye labeling.

(A) Sequence-independent assessment of unwinding preference. (i) Schematic of substrate Y15’ designed with identical sequences on both leading and lagging strands to eliminate sequence bias. (ii) Titration of ZRANB3 on DNA substrates (10 nM). (iii) Quantification of unwinding efficiency (n = 5; mean ± s.e.m.). **(B)** Fluorescent-dye swap control for single-molecule strand-displacement assays. (i) Substrate sY15 schematic with Cy5 (red) labeling the leading strand and Cy3 (green) labeling the lagging strand, reversing the dye positions from the original substrate (see Fig. 1F). (ii) Survival curves showing strand-displacement kinetics, confirming that dye-labeling does not affect strand unwinding preference (n > 1162 molecules).

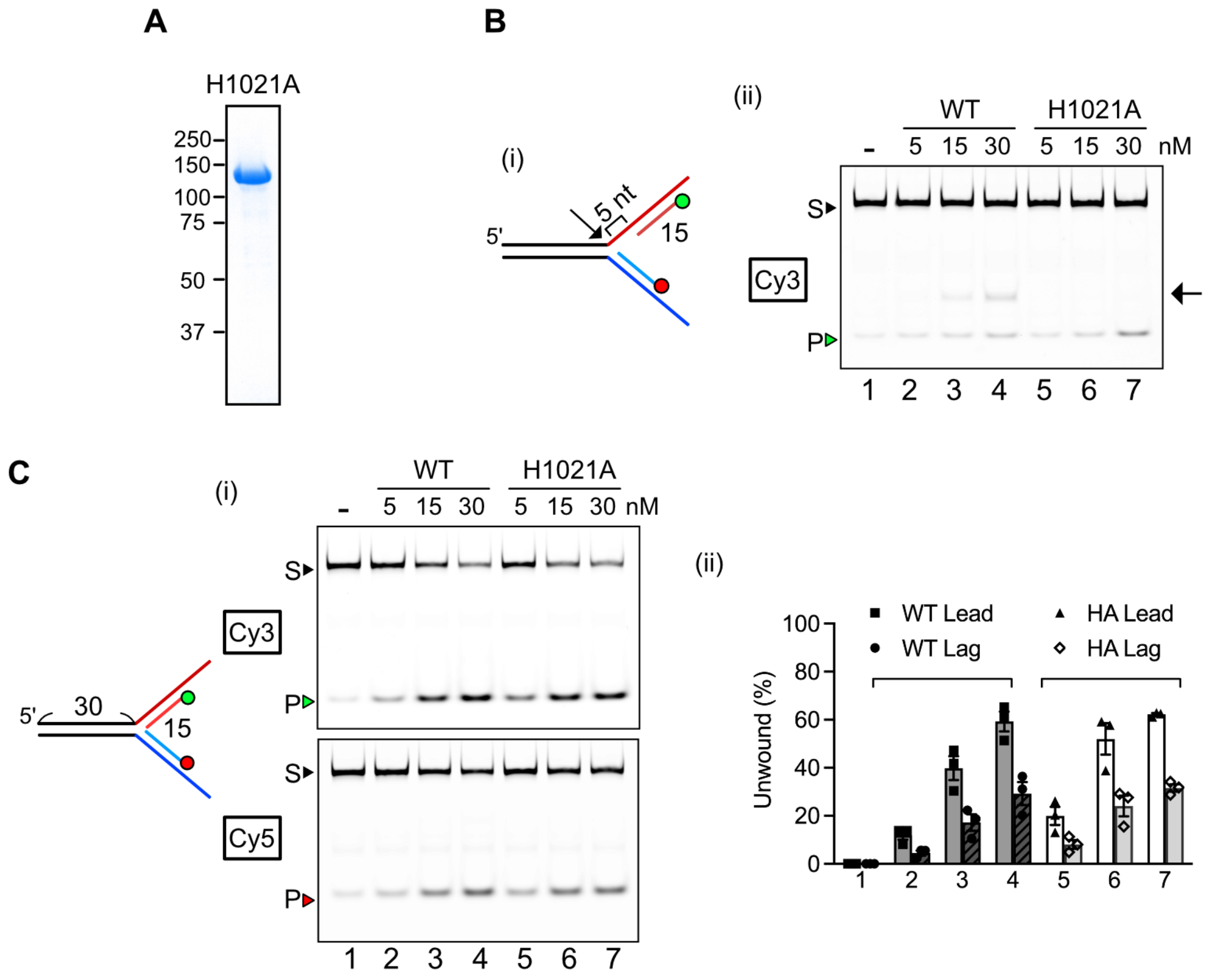

Supplementary Figure S3. Nuclease-dead ZRANB3 (H1021A) lacks endonuclease activity but retains unwinding activity.

(A) Coomassie-stained 10% SDS–PAGE gel showing purified nuclease-dead ZRANB3 (H1021A). **(B)** Loss of endonuclease activity for the H1021A mutant. (i) Schematic of the fork substrate bearing a 5-nt gap on the leading strand; the arrow marks the expected cleavage site for ZRANB3 endonuclease activity. (ii) Titration of wild-type (WT) or H1021A mutant protein on the 5-nt gap substrate (10 nM). Only WT protein exhibited cleavage activity (arrow), whereas the H1021A mutant showed no detectable nuclease activity. **(C)** The H1021A mutant retains unwinding activity. (i) Titration of WT or H1021A mutant protein on DNA substrate (10 nM) under the same conditions as described in (B). (ii) Quantification of unwound products shows comparable activity (n = 3; mean ± s.e.m.).

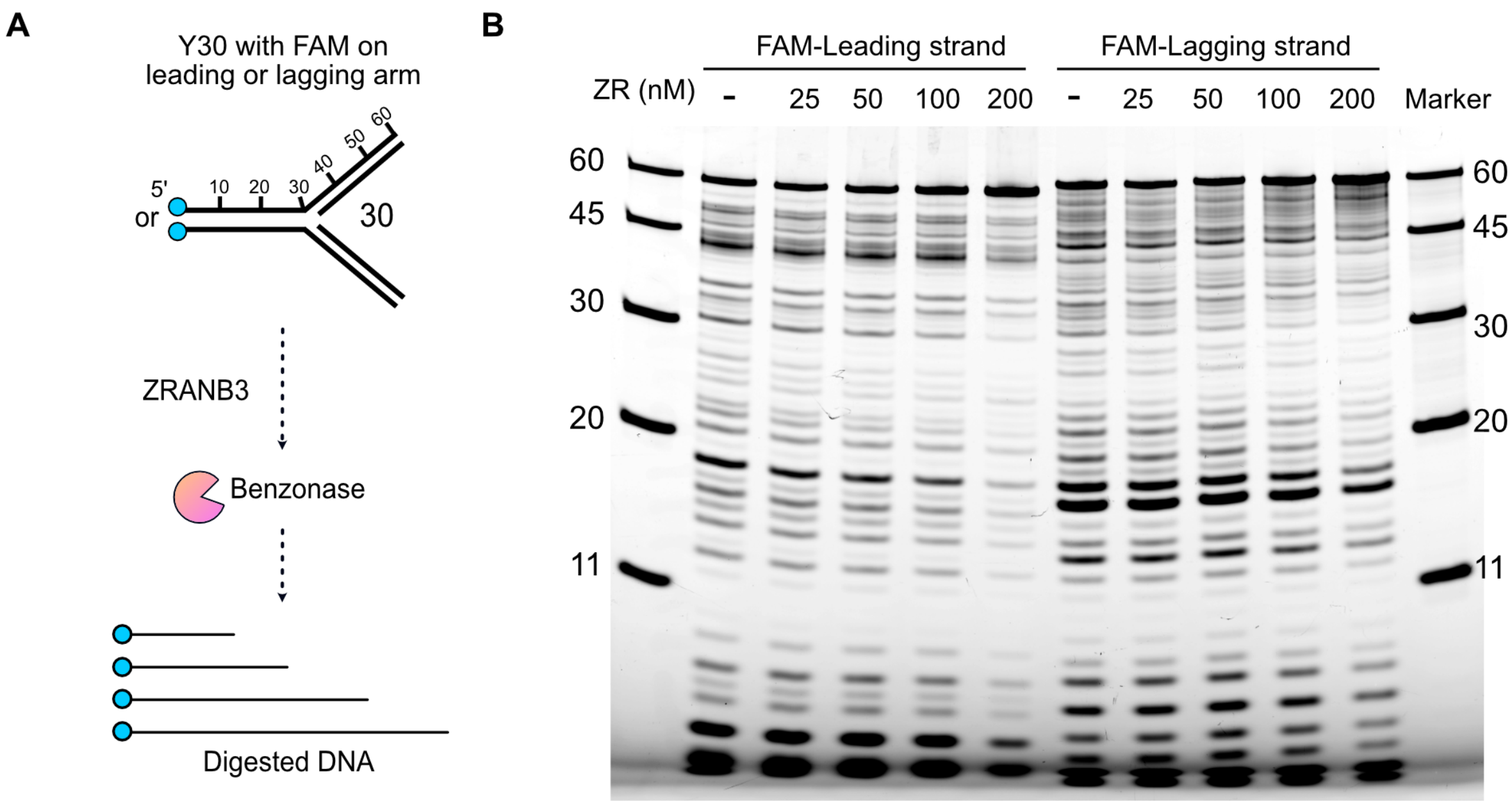

Supplementary Figure S4. Footprinting analysis of the ZRANB3-DNA complex.

(A) Schematic of the footprinting assay. Y30 fork DNA (50 nM), labeled with FAM on either the leading or lagging strand, was incubated with the indicated amount of ZRANB3 (up to 200 nM) at 37 °C for 20 min, followed by a 3-min digestion with Benzonase. Reactions were stopped and analyzed on a 27 % denaturing polyacrylamide gel. **(B)** Representative denaturing gel. Lanes with 0 nM and 100 nM ZRANB3 were used for the protection-ratio quantification in Fig. 3A. Results are representative of three independent experiments.

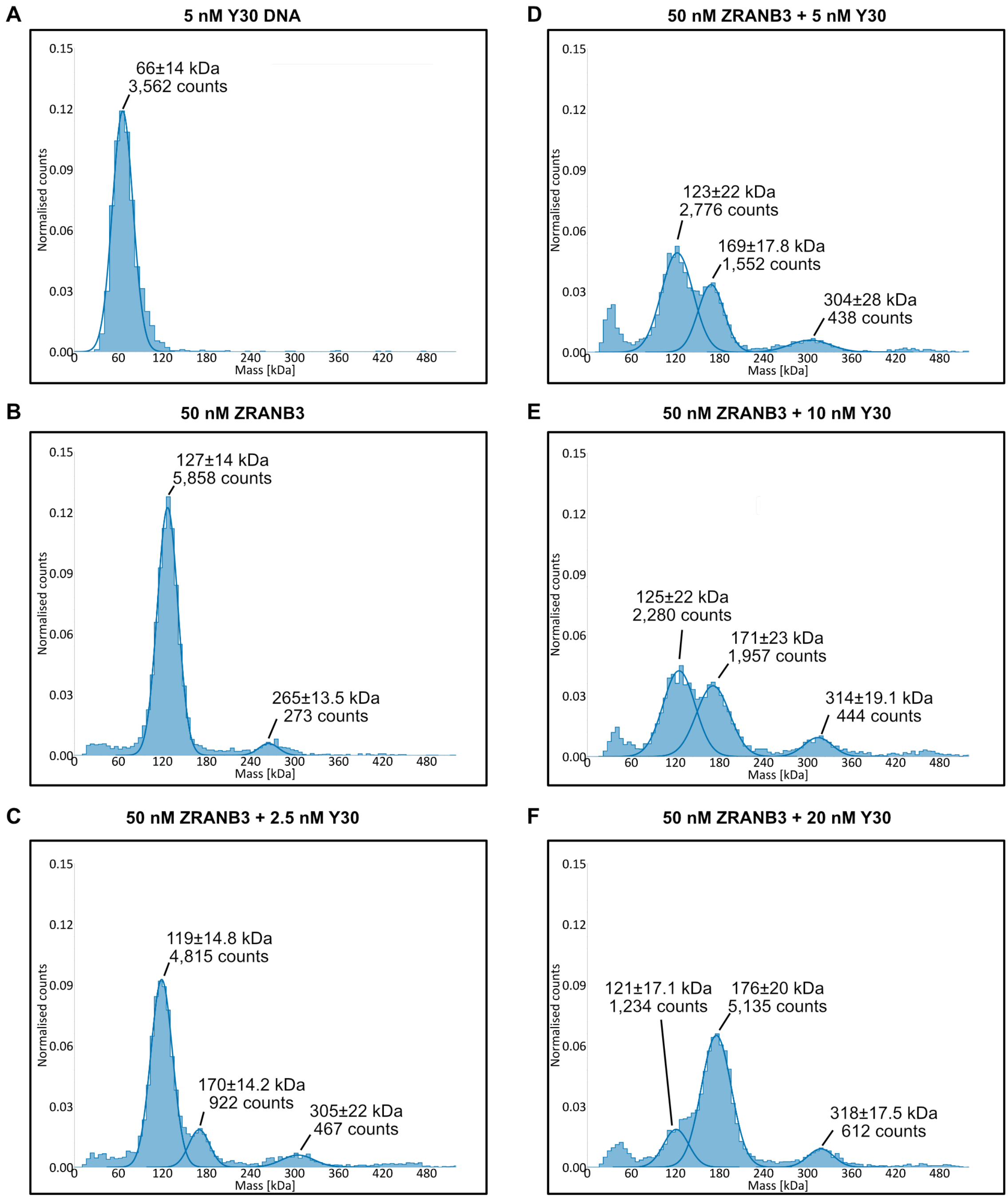

Supplementary Figure S5. Mass photometry spectra of ZRANB3-Y30 DNA complexes.

(A–F) ZRANB3 was incubated with the indicated concentrations of Y30 fork DNA and subjected to mass photometry. Spectra display peaks for free ZRANB3, free DNA, and 1:1 or 2:1 ZRANB3-DNA complexes, each labeled with its calculated molecular weight. The relative abundance of each species was quantified and is summarized in Fig. 3B.

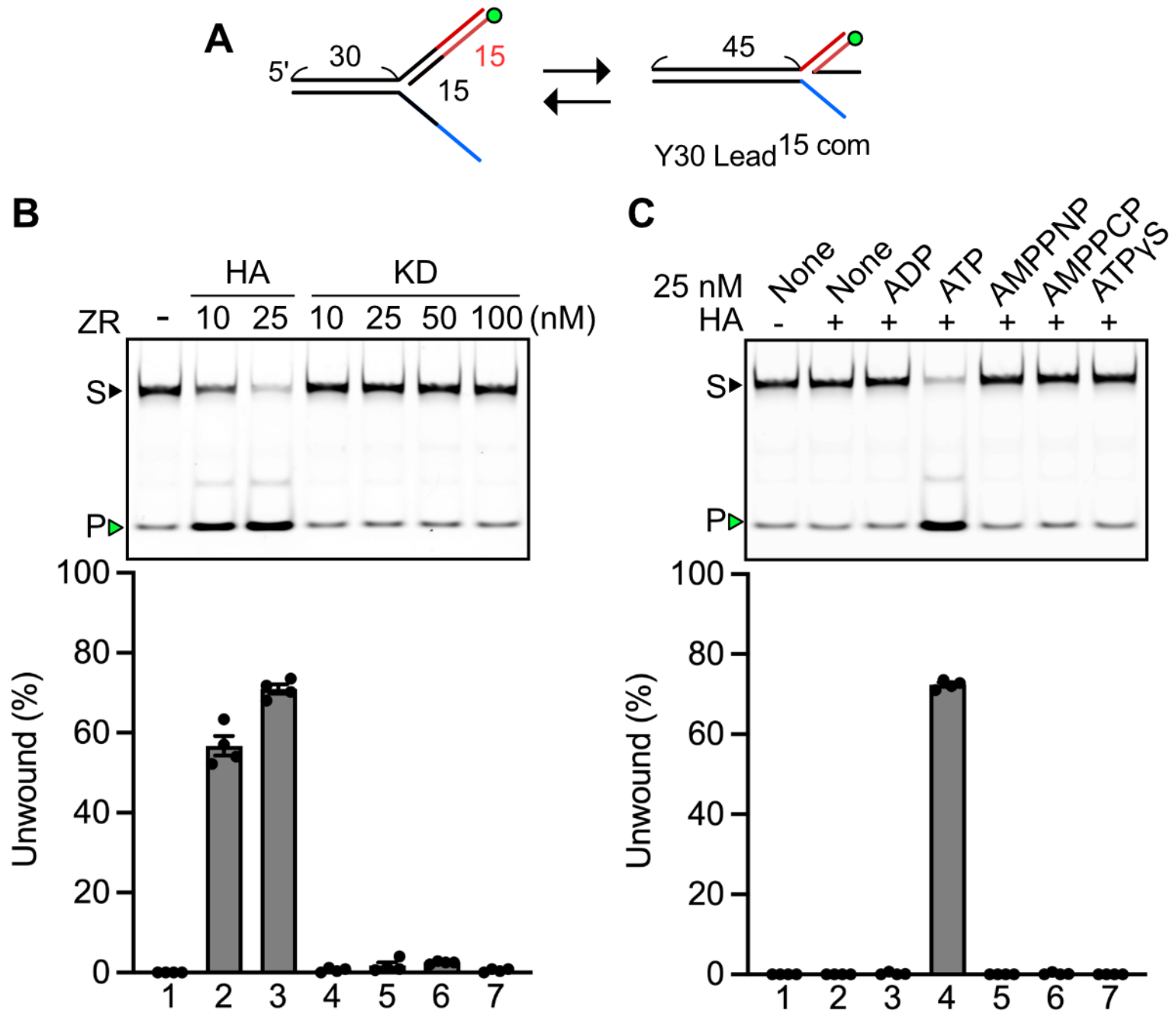

Supplementary Figure S6. ATP hydrolysis is required for ZRANB3-mediated unwinding during fork remodeling.

(A) Schematic of the Y30 Lead$^{15\ com}$ substrate. The first 15 nt from the fork junction on the leading strand is complementary to the parental strand (black), allowing a movable junction. The remaining unpaired regions on the splayed arms are shown in blue (lagging) and red (leading). **(B)** Representative unwinding assay using the Y30 Lead$^{15\ com}$ substrate (10 nM). The nuclease-dead H1021A mutant (HA) was used instead of wild-type ZRANB3 to prevent substrate cleavage. Titration of nuclease-dead H1021A or the ATPase-dead mutant K163D (KD) on DNA (10 nM). H1021A retains unwinding activity, whereas K163D does not (n = 4; mean ± s.e.m.). **(C)** H1021A was tested for its unwinding activity in the presence of ATP analogs (n = 4; mean ± s.e.m.).

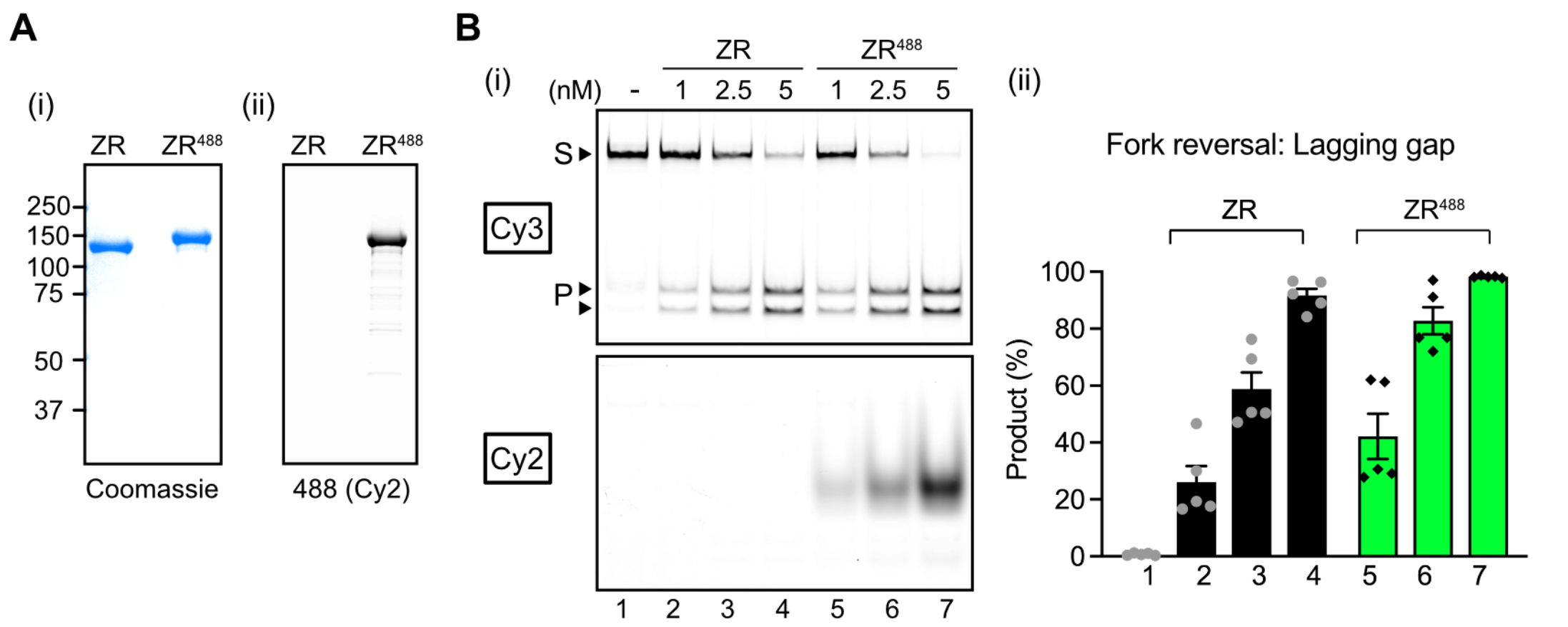

Supplementary Figure S7. Verification of SNAP-ZRANB3^{488} in catalyzing fork reversal.

(A) 10% SDS–PAGE gel showing purified ZRANB3 and SNAP-ZRANB3^{488} analyzed by Coomassie blue (i). The signal of Alexa Flour 488 on the labeled protein was detected via the Cy2 channel (ii). **(B)** Fork-reversal activity of ZRANB3 and ZRANB3^{488} on lagging-gap substrate (16 nM). (i) Titration of ZRANB3 or ZRANB3^{488} on DNA substrates. Fork reversal products were resolved by native PAGE and quantified. The Cy2 signals indicated the digested ZRANB3^{488} after PK/SDS treatment. (ii) Data represent mean ± s.e.m. from n = 5 independent experiments.

Supplementary table S1. **DNA substrates used in this study.** Blue and red colors indicate the noncomplementary regions on the splayed arms of the unwinding assay substrates, whereas black indicates the complementary regions. Green, red, and blue dots represent Cy3, Cy5, and FAM labels, respectively. The orange diamond denotes a biotin label.

DNA substrate		Composition
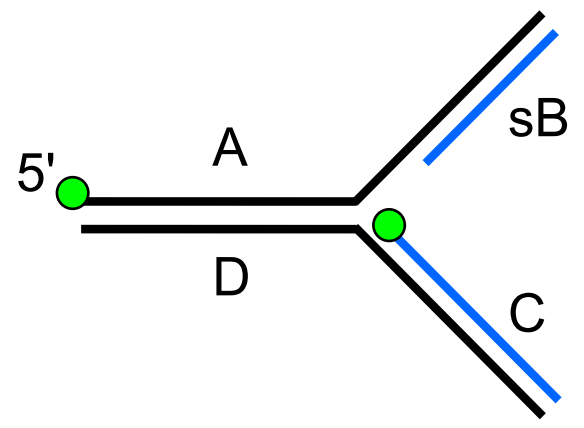	Leading gap fork [23]	1. A + sB and C + D<br>2. AsB + CD
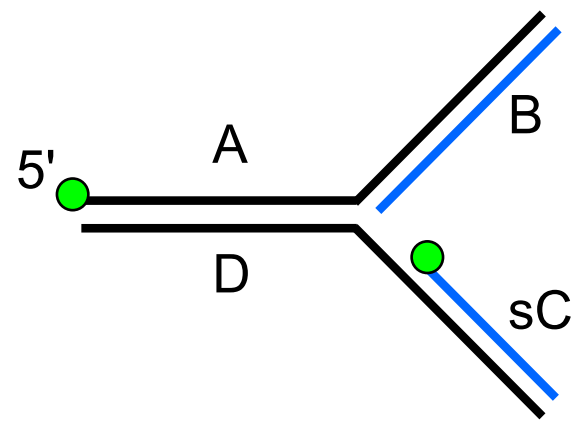	Lagging gap fork [23]	1. A + B and sC + D<br>2. AB + sCD
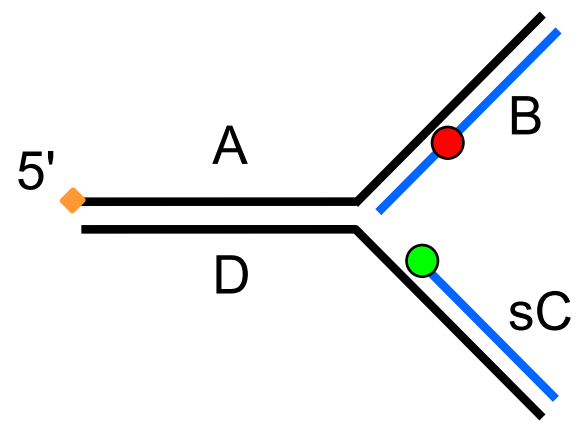	5'bio-Lagging gap fork (Single-molecule analysis in Fig. 5)	1. 5'bio-A + B and sC + D<br>2. 5'bio-AB + sCD
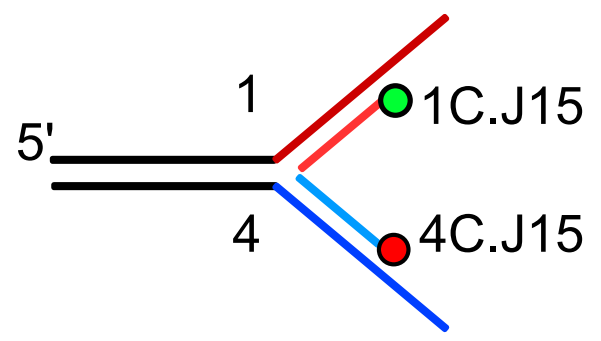	Y15	1 + 4 + 1C.J15 + 4C.J15
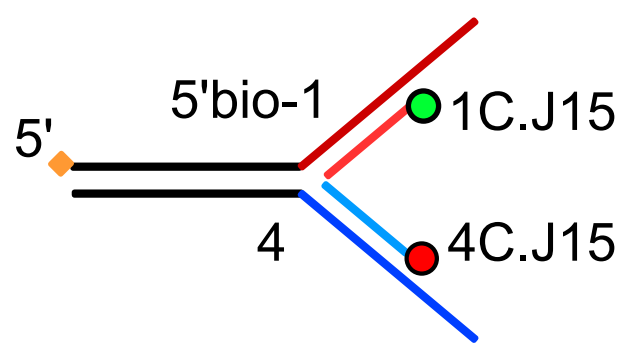	5'bio-Y15 (Single-molecule analysis in Fig. 1E)	5'bio-1 + 4 + 1C.J15 + 4C.J15
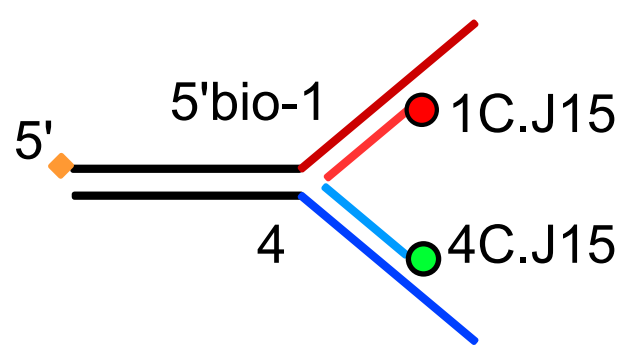	5'bio-sY15 (Single-molecule analysis in Supplementary Fig. S2B)	5'bio-1 + 4 + 1C.J15 + 4C.J15
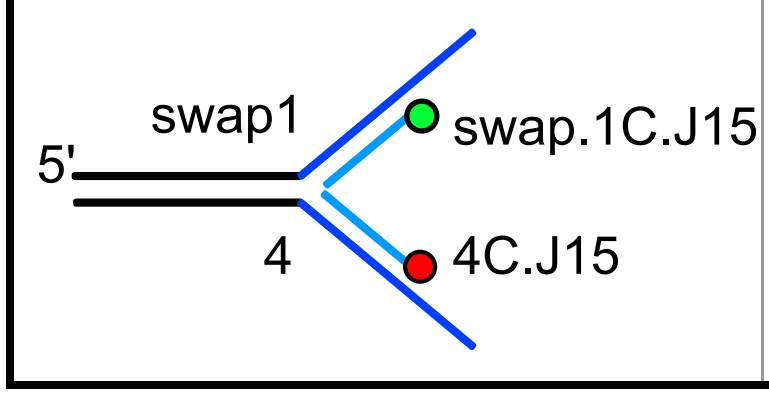	Y15'	swap1 + 4 + swap.1C.J15 + 4C.J15

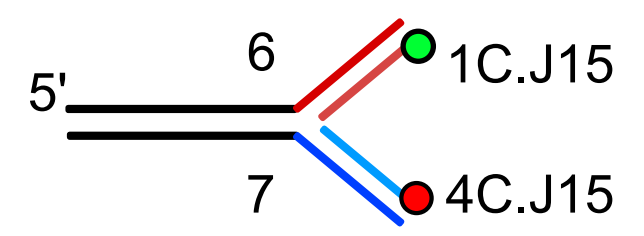	Y15 blunt	6 + 7 + 1C.J15 + 4C.J15
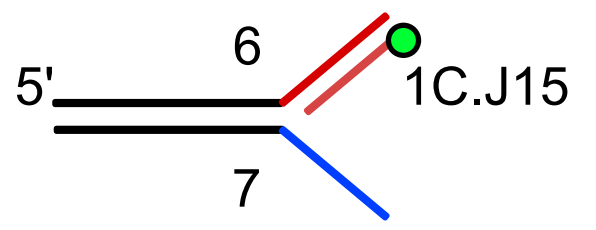	Y15 blunt lead	6 + 7 + 1C.J15
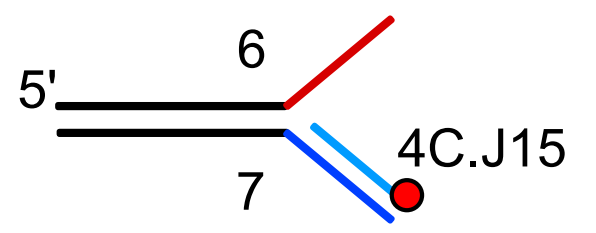	Y15 blunt lag	6 + 7 + 4C.J15
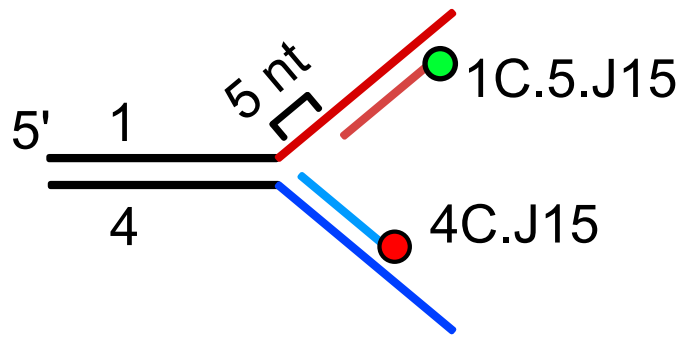	Y15 lead gap 5	1 + 4 + 1C.5.J15 + 4C.J15
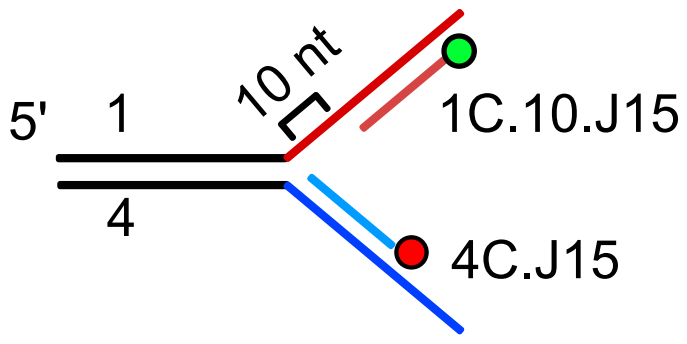	Y15 lead gap 10	1 + 4 + 1C.10.J15 + 4C.J15
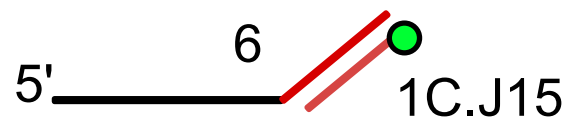	5' overhang	6 + 1C.J15
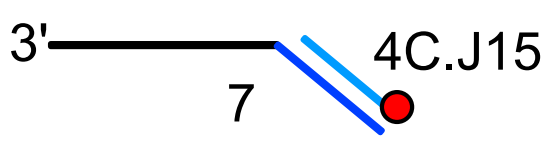	3' overhang	7 + 4C.J15
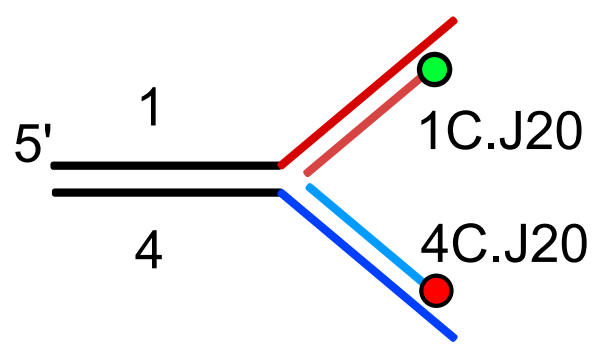	Y20	1 + 4 + 1C.J20 + 4C.J20
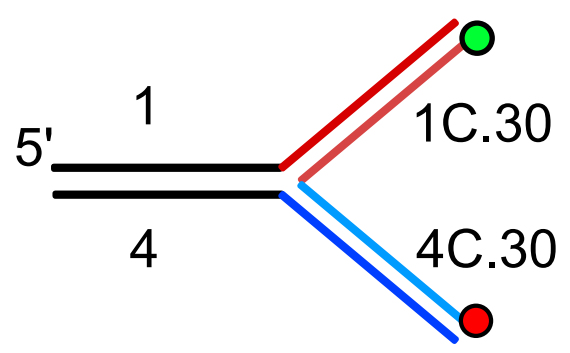	Y30	1 + 4 + 1C.30 + 4C.30
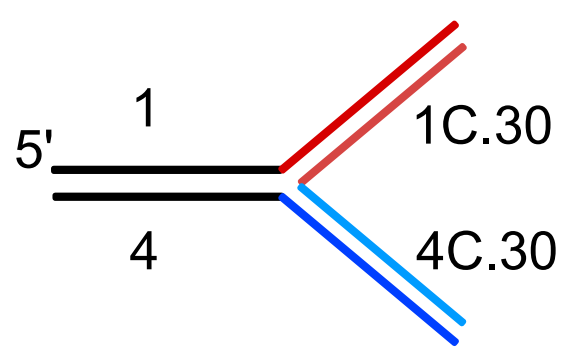	Y30 (Mass photometry and native MS)	Same oligo composition as Y30 but w/o fluorescence
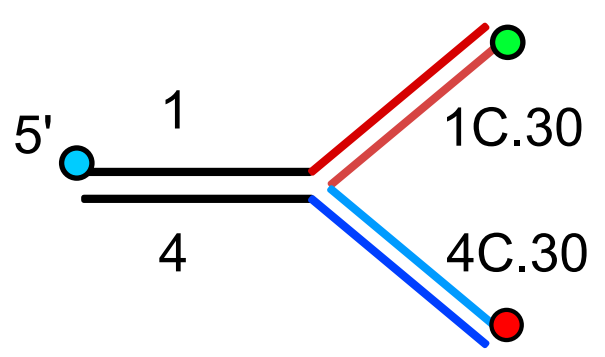	Y30 (Footprinting analysis FAM-Leading)	FAM-1 + 4 + 1C.30 + 4C.30

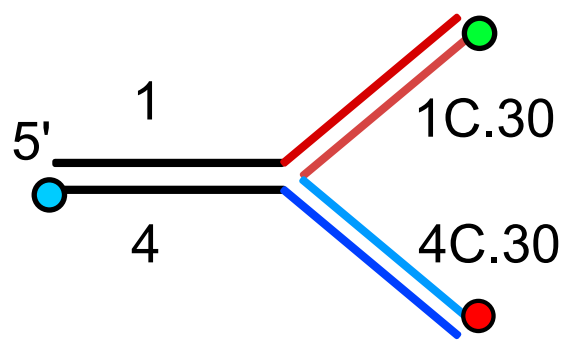	Y30<br>(Footprinting analysis FAM-Lagging)	1 + FAM-4 + 1C.30 + 4C.30
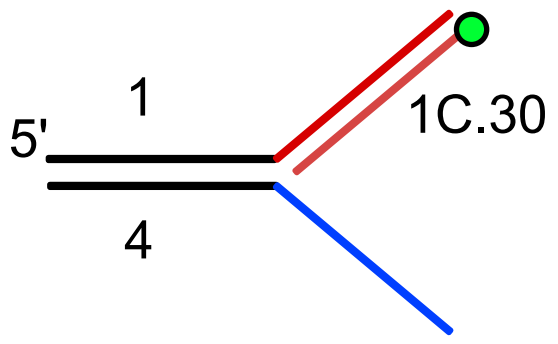	Y30 Lead	1 + 4 + 1C.30
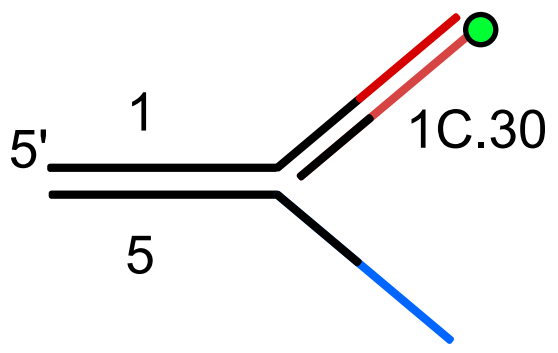	Y30 Lead$^{15\ \mathrm{com}}$	1 + 1C.30 + 5
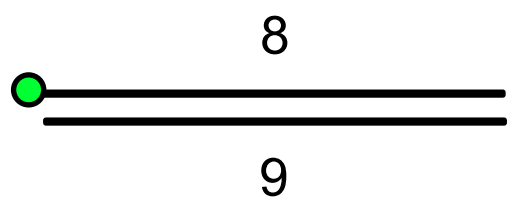	Annealing assay	8 + 9

Supplementary table S2. The DNA oligos for composing DNA substrates listed in Table S2.

Oligo	Length (nt)	Sequence (5'-3')
A	122	Cy3-CGTGACTTGATGTTAACCCTAACCCTAAGATATCGCGTTATCAGAGTGTGAGGATACATGTAGGCAATTGCCACGTGTCTATCAGCTGAAGTTGTTCGCGACGTGCGATCGTCGCTGCGACG
5'bio-A	122	Bio-CGTGACTTGATGTTAACCCTAACCCTAAGATATCGCGTTATCAGAGTGTGAGGATACATGTAGGCAATTGCCACGTGTCTATCAGCTGAAGTTGTTCGCGACGTGCGATCGTCGCTGCGACG
sB	52	CGTCGCAGCGACGATCGCACGTCGCGAACAACTTCAGCTGATAGACACGTGG
B	82	CGTCGCAGCGACGATCGCACGTCGCGAACAACTTCAGCTGATAGACACGTGGCAATTGCCTACATGTATCCTCACACTCTGA
iB	82	CGTCGCAGCGACGATCGCACGTCGCGAACAACTTCAGCTGATAGACACGT/Cy5/GGCAATTGCCTACATGTATCCTCACACTCTGA
C	82	Cy3-TCAGAGTGTGAGGATACATGTAGGCAATTGCCACGTGTCTATCAGCTGAAGTTGTTCGCGACGTGCGATCGTCGCTGCGACG
sC	52	Cy3-CCACGTGTCTATCAGCTGAAGTTGTTCGCGACGTGCGATCGTCGCTGCGACG
D	122	CGTCGCAGCGACGATCGCACGTCGCGAACAACTTCAGCTGATAGACACGTGGCAATTGCCTACATGTATCCTCACACTCTGAATACGCGATATCTTAGGGTTAGGGTTAACATCAAGTCACG
1	60	ACGCTGCCGAATTCTACCAGTGCCTTGCTAGGACATCTTTGCCCACCTGCAGGTTCACCC
5'bio-1	60	Bio-ACGCTGCCGAATTCTACCAGTGCCTTGCTAGGACATCTTTGCCCACCTGCAGGTTCACCC
FAM-1	60	FAM-ACGCTGCCGAATTCTACCAGTGCCTTGCTAGGACATCTTTGCCCACCTGCAGGTTCACCC

4	60	CGATAGTCGGATCCTCTAGACAGCTCCATGTAGCAAGGCACTGGTAGAATTCGGCAGCGT
FAM-4	60	CGATAGTCGGATCCTCTAGACAGCTCCATGTAGCAAGGCACTGGTAGAATTCGGCAGCGT-FAM
Swap1	60	ACGCTGCCGAATTCTACCAGTGCCTTGCTAGTACCTCGACAGATCTCCTAGGCTGATAGC
5	60	CGATAGTCGGATCCTTGGGCAAAGATGTCCTAGCAAGGCACTGGTAGAATTCGGCAGCGT
6	45	ACGCTGCCGAATTCTACCAGTGCCTTGCTAGGACATCTTTGCCCA
7	45	CTAGACAGCTCCATGTAGCAAGGCACTGGTAGAATTCGGCAGCGT
1C.J15	15	Cy3-TGGGCAAAGATGTCC
1C.J15	15	Cy5-TGGGCAAAGATGTCC
swap.1C.J15	15	Cy3-GATCTGTCGAGGTAC
4C.J15	15	CATGGAGCTGTCTAG-Cy5
4C.J15	15	CATGGAGCTGTCTAG-Cy3
1C.J20	20	Cy3-GCAGGTGGGCAAAGATGTCC
4C.J20	20	CATGGAGCTGTCTAGAGGAT-Cy5
1C.30	30	Cy3-GGGTGAACCTGCAGGTGGGCAAAGATGTCC
4C.30	30	CATGGAGCTGTCTAGAGGATCCGACTATCG-Cy5
1C.5.J15	15	Cy3-GCAGGTGGGCAAAGA
1C.10.J15	15	Cy3-AACCTGCAGGTGGGC
8	53	Cy3-TCCTTTTGATAAGAGGTCATTTTTGCGGATGGCTTAGAGCTTAATTGCTGAAT
9	53	ATTCAGCAATTAAGCTCTAAGCCATCCGCAAAAATGACCTCTTATCAAAAGGA
FAM.1.11	11	FAM-ACGCTGCCGAA
FAM.1.20	20	FAM-ACGCTGCCGAATTCTACCAG
FAM.1.30	30	FAM-ACGCTGCCGAATTCTACCAGTGCCTTGCTA
FAM.1.45	45	FAM-ACGCTGCCGAATTCTACCAGTGCCTTGCTAGGACATCTTTGCCCA
FAM.4.11	11	TTCGGCAGCGT-FAM

FAM.4.20	20	CTGGTAGAATTCGGCAGCGT-FAM
FAM.4.30	30	TAGCAAGGCACTGGTAGAATTCGGCAGCGT-FAM
FAM.4.45	45	CTAGACAGCTCCATGTAGCAAGGCACTGGTAGAATTCGGCAGCGT-FAM

Supplementary table S3. Molecular masses of ZRANB3 and DNA complexes as measured by native MS.

	m/z value	Observed mass (Da)	Theoretical mass (Da)	ΔMass (Da)
**Y30 DNA**	13+ 4260.41<br>12+ 4615.35<br>11+ 5035.65	55375 ± 5	55368	7, 0.0%
**ZRANB3**	23+ 5499.56<br>22+ 5749.25<br>21+ 6023.23<br>20+ 6324.53	126466 ± 4	126217	249, 0.2%
**ZRANB3-Y30 complex (1:1)**	27+ 6735.69<br>26+ 6995.33<br>25+ 7273.86<br>24+ 7577.22<br>23+ 7907.03<br>22+ 8267.55	181840 ± 16	181585	255, 0.1%
**ZRANB3-Y30 complex (2:1)**	36+ 8567.05<br>35+ 8812.19<br>34+ 9071.73<br>33+ 9346.07	308390 ± 11	307802	588, 0.2%
